# Proposal of Two New Combinations, Twenty New Species, Four New Genera, One New Family, and One New Order for the Anamorphic Basidiomycetous Yeast Species in *Ustilaginomycotina*

**DOI:** 10.3389/fmicb.2021.777338

**Published:** 2022-02-11

**Authors:** Yao-Yao Li, Man-Man Wang, Marizeth Groenewald, Ai-Hua Li, Yun-Tong Guo, Feng Wu, Bing-Qian Zhang, Eiji Tanaka, Qi-Ming Wang, Feng-Yan Bai, Dominik Begerow

**Affiliations:** ^1^Engineering Laboratory of Microbial Breeding and Preservation of Hebei Province, School of Life Sciences, Institute of Life Sciences and Green Development, Hebei University, Baoding, China; ^2^Westerdijk Fungal Biodiversity Institute, Utrecht, Netherlands; ^3^China General Microbiological Culture Collection Center and State Key Laboratory of Microbial Resources, Institute of Microbiology, Chinese Academy of Sciences, Beijing, China; ^4^Department of Environmental Science, Ishikawa Prefectural University, Nonoichi, Japan; ^5^State Key Laboratory of Mycology, Institute of Microbiology, Chinese Academy of Sciences, Beijing, China; ^6^Department of Evolution of Plants and Fungi, Ruhr-Universität Bochum, Bochum, Germany

**Keywords:** *Ustilaginomycotina*, yeasts, smuts, 28 new taxa, molecular phylogeny

## Abstract

Two hundred and forty-four ustilaginomycetous yeast or yeast-like strains were isolated from the soil, skin of animals or humans and plant materials during the past 20 years. Among them, 203 strains represent 39 known species, whereas 41 strains represent several novel species based on the sequence analyses of the rDNA genes [18S rDNA, Internal Transcribed Spacer (ITS) regions, 26S rDNA D1/D2 domain] and three protein genes (*RPB1, RPB2*, and *TEF1*). In this study, one new order, one new family, four new genera, twenty new species, and two new combinations were proposed. They are *Franziozymales* ord. nov., *Franziozymaceae* fam. nov., *Baueromyces* gen. nov., *Franziozyma* gen. nov., *Guomyces* gen. nov., *Yunzhangomyces* gen. nov., *Baueromyces planticola* sp. nov., *Franziozyma bambusicola* sp. nov., *Gjaerumia cyclobalanopsidis* sp. nov., *Gjaerumia pseudominor* sp. nov., *Jamesdicksonia aceris* sp. nov., *Jaminaea lantanae* sp. nov., *Kalmanozyma hebeiensis* sp. nov., *Langdonia ligulariae* sp. nov., *Meira hainanensis* sp. nov., *Meira pileae* sp. nov., *Meira plantarum* sp. nov., *Phragmotaenium parafulvescens* sp. nov., *Sporisorium cylindricum* sp. nov., *Sympodiomycopsis europaea* sp. nov., *Tilletiopsis lunata* sp. nov., *Tilletiopsis pinicola* sp. nov., *Yunzhangomyces clavatus* sp. nov., *Yunzhangomyces cylindricus* sp. nov., *Yunzhangomyces qinlingensis* sp. nov., *Yunzhangomyces orchidis* sp. nov., *Guomyces nicotianae* comb. nov., and *Yunzhangomces scirpi* comb. nov.

## Introduction

The subphylum *Ustilaginomycotina* (*Basidiomycota, Fungi*) comprises a variety of lifestyles. The majority of species are biotrophic pathogens known as smuts, whereas some anamorphic yeast lineages are saprotrophs or, possibly, mycoparasites (Begerow et al., 2000, 2014, 2017; Bauer et al., 2001; Wang et al., 2014, 2015). The asexual yeast species were placed in nine genera, namely, *Acaromyces* (Boekhout et al., 2003), *Farysizyma* (Inácio et al., 2008), *Jaminaea* (Sipiczki and Kajdacsi, 2009), *Malassezia* (Boekhout et al., 2003), *Meira* (Boekhout et al., 2003), *Moniliella* (Stolk and Dakin, 1966), *Pseudozyma* (Boekhout, 1995), *Sympodiomycopsis* (Sugiyama et al., 1991), and *Tilletiopsis* (Gokhale, 1972; Boekhout, 1991; Boekhout et al., 1995), and reclassified in four classes in the *Ustilaginomycotina*, namely, *Exobasidiomycetes*, *Malasseziomycetes*, *Moniliellomycetes*, and *Ustilaginomycetes* (Bauer et al., 2001; Hibbett et al., 2007; Begerow et al., 2014; Wang et al., 2014). Most of these yeast genera are monophyletic, however, the genera *Pseudozyma* and *Tilletiopsis* were polyphyletic (Begerow et al., 2000, 2006, 2014; Fell et al., 2000; Stoll et al., 2003, 2005; Boekhout et al., 2011; McTaggart et al., 2012a,b). Recently, those two anamorphic polyphyletic genera were revised by Wang et al. (2015) based on multigene phylogenetic analyses. Except *Pseudozyma alboarmeniaca pro tem.*, *P. thailandica pro tem.*, *P. tsukubaensis pro tem.*, *P. hubeiensis pro tem.*, and *P. pruni pro tem.*, the other species of *Pseudozyma* were transferred to *Ustilago, Moesziomyces*, *Triodiomyces*, *Sporisorium*, *Langdonia, Kalmanozyma*, and *Dirkmeia*, and the genus *Pseudozyma* was treated as a synonym of *Ustilago* (Wang et al., 2015). Three species were kept in the emended genus, *Tilletiopsis*, while the others were transferred to the genera *Phragmotaenium*, *Gjaerumia*, *Robbauera*, and *Golubevia*. *Rhodotorula bacarum* was treated as a synonym of *Microstroma album*, while *Rhodotorula hinnulea* and *Rhodotorula phylloplana* were synonymized and transferred to *Microstroma* as one single species *Microstroma phylloplanum* (Wang et al., 2015). Then, it was transferred to a newly described anamorphic genus *Pseudomicrostroma* by Kijpornyongpan and Aime (2017). All species in *Farysizyma* have been transferred to the teleomorphic genus, *Farysia* (Wang et al., 2015).

Benefiting from the molecular phylogenetic analyses, a large number of asexual fungi, especially at genus and higher ranks in *Ustilaginomycotina* were discovered (Begerow et al., 2014). Recently, three monotypic asexual genera in *Ustilaginomycotina* were proposed (Nasr et al., 2014; Albu et al., 2015; Sun et al., 2018). The genus *Fereydounia* represents the first yeast species in *Urocystidales* (Nasr et al., 2014). *Violaceomyces* is a yeast-like fungus in *Violaceomycetales* (Albu et al., 2015) and *Capitulocladosporium* is a *Cladosporium*-like fungus, but phylogenetically related to *Violaceomycetales* and *Uleiellales* in *Ustilaginomycetes* (Sun et al., 2018). Six *Tilletiopsis*-like yeast novel species in *Exobasidiomycetes* were described based on the phylogenetic analyses of multi-loci and LSU rDNA by Richter et al. (2019).

Over the past 22 years, more than 1,500 basidiomycetous yeast strains isolated from the soil, skin of animals or humans, and plant materials have been identified by analyzing the D1/D2 domain of the ribosomal large subunit DNA (D1/D2) and the ITS sequences in the State Key Laboratory of Mycology, China. Most of them belonging to *Agaricomycotina* and *Pucciniomycotina*. In addition, eight new genera, three families, and two orders have been documented in the article published by Li et al. (2020). In this study, a similar approach undertaken by Li et al. (2020) was used to propose one new order, one new family, four new genera, twenty new species, and two new combinations in the *Ustilaginomycotina*.

## Materials and Methods

### Strain Sampling and Phenotype Analyses

The yeast or yeast-like strains studied are listed in Table 1. Strains were isolated from plant leaves by using the improved ballistoconidia-fall method proposed by Nakase and Takashima (1993) and from the soil, tree bark, and rotten wood by an enrichment method described by Li et al. (2020). Yeasts were isolated from the skin of humans and animals by using the following protocol. The samples from human faces and heads and the skin of animals were collected with sterile swabs. Swabs were gently rolled back-and-forth 2–4 times across the skin and were then streaked onto Leeming and Notman agar plates (Leeming and Notman, 1987). The phenotypic and biochemotaxonomic characters were examined according to the methods introduced by Kurtzman et al. (2011). The sexual test and the ballistoconidium-forming activity of all the new species were investigated as described by Li et al. (2020).

**TABLE 1:** List of yeast or yeast-like species employed and GenBank numbers determined in this study.

Species	Holotype	CBS collection	Laboratory strain			18S + ITS + D1/D2	RPB1	RPB2	EF1
***Ustilaginaceae* (*Ustilaginales, Ustilaginomycetes*)**									
*Sporisorium cylindricum* sp. nov.	CGMCC 2.3756	CBS 15755	WZS28.2B	Wuzhishan Montain, Hainan province, China, Octomber 2007	Leaf of unidentified plant	MN901699	MN901756	MN901781	MN901669
	CGMCC 2.3576		YX3.6	Kunming country, Yunnan province, China, May 2007	leaf of unidentified plant	MN901698	/	/	MN901666
*Kalmanozyma hebeiensis* sp. nov.	CGMCC 2.3457	CBS 15483	H8A4	Hebei province, China, April 2007	leaf of unidentified plant	MN901700	/	MN901775	MN901662
*Langdonia walkerae*	CGMCC 2.4680	CBS 144911	JX1243	China, Sptember 2012	leaf of unidentified plant	MN901702	/	MN901787	MN901675
*Langdonia ligulariae* sp. nov.	CGMCC 2.6313	CBS 15581	XZ146B3	Lulang county, Tibet, China, Sptember 2014	leaf of *Ligularia tsangchanensis*	MN901697	MN901766	/	MN901684
***Brachybasidiaceae* (*Exobasidiales, Exobasidiomycetes*)**									
*Meira plantarum* sp. nov.	CGMCC 2.4430	CBS 12491	FJYZ8-3	Fuzhou county, Fujian province, China, August 2011	Leaf of unidentified plant	MN901704	MN901757	MN901782	MN901670
	CGMCC 2.4432		FJYZ11-7	Fuzhou county, Fujian province, China, August 2011	Leaf of unidentified plant	MT896142	/	/	/
	CGMCC 2.4431		FJYZ5-8	Fuzhou county, Fujian province, China, August 2011	Leaf of unidentified plant	MT896141	/	/	/
	CGMCC 2.6306	CBS 144914	XZ123E33	Beibengxiang, Motuo county, Tibet, China, Sptember 2014	Leaf of unidentified plant	MT896139	MN901764	/	MN901682
*Meira pileae* sp. nov.	CGMCC 2.6305	CBS 144915	XZ123B4	Beibengxiang, Motuo county, Tibet, China, Sptember 2014	Leaf of *Pilea* sp.	MT896138	/	MN901791	MN901681
*Meira hainanensis* sp. nov.	CGMCC 2.3537	CBS 15497	WZS12.12	Wuzhishan Montain, Hainan province, China, May 2007	Leaf of unidentified plant	MN901703	MN901753	MN901777	MN901664
*Yunzhangomyces orchidis* sp. nov.	CGMCC 2.3451	CBS 15753	WZS24.28	Wuzhishan Montain, Hainan province, China, Apir 2007	Leaf of Orchidaceae	MN901726	/	/	MN901661
*Yunzhangomyces clavatus* sp. nov.	CGMCC 2.4433	CBS 144908	FJYZ8-4	Fuzhou country, Fujian province, China, August 2011	Leaf of unidentified plant	MN901724	MN901758	MN901783	MN901671
		CBS 144917	XZ128D	Heilongxiang, Motuo county, Tibet, China, Sptember 2014	Leaf of *Impatiens* sp.	MN901725	MN901765	MN901792	MN901683
*Yunzhangomyces qinlingensis* sp. nov.	CGMCC 2.4533	CBS 144910	ZHH5D15	Qinling, Heihe, Shaanxi province, China March 2012	Leaf of unidentified plant	MN901729	MN901759	MN901785	MN901673
*Yunzhangomyces cylindricus* sp. nov.	CGMCC 2.6304	CBS 15585	HLJ17B1	Daliangzi river national forest park, Heilongjiang province, China, August 2015	Leaf of unidentified plant	MN901728	/	MN901790	MN901680
*Yunzhangomyces* sp.			HLJ9.21	Daliangzi river national forest park, Heilongjiang province, China, August 2015	Leaf of unidentified plant	MN901727	/	/	/
***Georgefischeriales* (*Exobasidiomycetes*)**									
*Phragmotaenium parafulvescens* sp. nov.	CGMCC 2.3573	CBS 15754	SY9.2	Sanya country, Hainan province, China, May 2007	Leaf of unidentified plant	MN901716	MN901754	MN901778	MN901665
*Gjaerumia pseudominor* sp. nov.	CGMCC 2.5616	CBS 144912	TY1AS	Heilongjiang province, China, August 2015	Leaf of unidentified plant	MN901705	MN901761	MN901789	MN901677
*Gjaerumia cyclobalanopsidis* sp. nov.	CGMCC 2.6419	CBS 144918	GT31C4	Gutianshan, Zhejiang province, China, June 2011	Leaf of *Cyclobalanopsis* sp.	MT896140	/	/	/
*Jamesdicksonia aceris* sp. nov.	CGMCC 2.5679	CBS 144913	HLJ11A4A	Heilongjiang province, China, August 2015	Leaf of unidentified plant	MN901731	/	MN901796	MN901687
	CGMCC 2.2370	CBS 144907	CB297	Changbai mountain, Jilin province, October 1998	Leaf of unidentified plant	MN901732	/	MN901795	MN901686
	CGMCC 2.5602	CBS 144916	XZ155C4	Bomi, Tibet, China, Sptember 2014	Leaf of *Acer pectinatum*	MN901734	/	MN901793	MN901685
			XZ156C4	Bomi, Tibet, China, Sptember 2014	Leaf of unidentified plant	MN901735/MN901736	/	/	/
***Entylomatales* (*Exobasidiomycetes*)**									
*Tilletiopsis pinicola* sp. nov.	CGMCC 2.5613	CBS 15775	CBS 15775	Heilongjiang province, China, August 2015	A leaf of *Pinus* sp.	MN901708	MN901760	MN901788	MN901676
*Tilletiopsis lunata* sp. nov.	CGMCC 2.6308	DSM 111865	HE6AB1	Huzhong, Heilongjiang Province, China, August 2015	Leaf of unidentified plant	MN901706	MN901763	/	MN901679
	CGMCC 2.6307	DSM 111865	HE2A5	Huzhong, Heilongjiang Province, China, August 2015	Leaf of unidentified plant	MN901707	MN901762	/	MN901678
***Microstromatales* (*Exobasidiomycetes*)**									
*Jaminaea lantanae* sp. nov.	CGMCC 2.3529	HE2A5	HK13.4	Haikou country, Hainan province, China, May 2007	Leaf of *Lantana camara*	MN901709	/	MN901776	MN901663
	CGMCC 2.3622		HK13.4-2	Haikou country, Hainan province, China, May 2007	Leaf of *Lantana camara*	MN901710	/	MN901779	MN901667
*Sympodiomycopsis europaea* sp. nov.	CGMCC 2.3119	CBS 15470	G1.1	Germany, March 2006	Leaf of unidentified plant,	MN901717	MN901748	MN901771	MN901657
	CGMCC 2.3181		G7.21	Germany, March 2006	Leaf of unidentified plant,	MN901718	MN901752	MN901774	MN901660
	CGMCC 2.3120		G4.3	Germany, March 2006	Leaf of unidentified plant,	MN901719	/	/	/
	CGMCC 2.3121		G7.1-3	Germany, March 2006	Leaf of unidentified plant,	MN901720	MN901749	MN901772	MN901658
	CGMCC 2.3122		G7.2-2	Germany, March 2006	Leaf of unidentified plant,	MN901721	/	/	/
	CGMCC 2.3123		G11.2	Germany, March 2006	Leaf of unidentified plant,	MN901722	/	/	/
	CGMCC 2.3124		G16	Germany, March 2006	Leaf of unidentified plant,	MN901723	/	/	/
*Baueromyces planticola* sp. nov.	CGMCC 2.4532	CBS 144909	XS9B4	Xingshan country, Hubei province, China, March 2012	Leaf of unidentified plant	MN901712	/	MN901784	MN901672
	CGMCC 2.4534		GZMT1C2	Maotai county, Guizhou province	Leaf of unidentified plant	MN901713	/	MN901786	MN901674
	CGMCC 2.4535		FJS8A1	Fanjingshan, Guizhou province	Leaf of unidentified plant	MN901714	/	/	/
	CGMCC 2.4536		FJS8A1B	Fanjingshan, Guizhou province	Leaf of unidentified plant	MN901715	/	/	/
***Franziozymales* (*Exobasidiomycetes*)**									
*Franziozyma bambusicola* sp. nov.	CGMCC 2.2620	CBS 15774	XZ4C4	Bomi county, Tibet, China, Sptember 2004	A leaf of bamboo	MK415411	MK415413	MK415414	MK415412
			XZ4A1	Bomi county, Tibet, China, Sptember 2004	A leaf of bamboo	MZ045837	/	/	/

### PCR and DNA Sequencing

Deoxyribonucleic acid (DNA) was extracted following the method proposed by Wang and Bai (2008). The 18S (SSU) rDNA sequences were amplified according to Wang et al. (2003). The ITS (including the 5.8S rDNA) and 26S (LSU) rDNA D1/D2 regions were sequenced using the methods described previously (Wang and Bai, 2004). Amplification reactions and sequencing of the three protein genes, namely, two RNA polymerase II subunits (*RPB1* and *RPB2*) and the translation elongation factor 1-α (*TEF1*), were performed as described in Wang et al. (2014). GenBank sequence accession numbers determined during this study are listed in Table 1.

### Molecular Phylogenetic Analyses

Sequence alignments were performed with the MAFFT algorithm (Katoh and Standley, 2013) using the G-INS-i algorithm. The model GTR + I + G, the best nucleotide substitution model determined in MEGA 7.0 (Kumar et al., 2016), was selected for Bayesian inference (BI) and Maximum likelihood (ML) analyses. BI analysis was carried using MrBayes 3.1.2 (Ronquist et al., 2012) with the parameter settings proposed by Wang et al. (2015). ML phylogenetic reconstruction was performed using RAxML-HPC 7.2.8 (Stamatakis, 2006) with 500 bootstrap replicates. A Bayesian posterior probability (PP) of ≥0.9 or a bootstrap percentage (BP) of ≥70% was set as significantly supported in the constructed trees. The new alignments and trees in this study were deposited in TreeBASE (Nos. S28175).

## Results and Discussion

### Diversity and Ecology

Two hundred and forty-four ustilaginomycetous yeast or yeast-like strains isolated from soil (20%, 49/244), the skin of animals or humans (11%, 27/244), and plant materials (69%, 168/244), including leaves, tree bark, and rotten wood, were identified as 39 known species distributed in 15 genera, i.e., *Entyloma*, *Exobasidium*, *Gjaerumia*, *Golubevia*, *Langdonia, Meira*, *Moesziomyces*, *Mycosarcoma*, *Phragmotaenium*, *Pseudozyma pro. tem*, *Quambalaria*, *Robbauera*, *Sporisorium*, *Tilletiopsis*, and *Ustilago*, and 20 undescribed species (Table 1 and Supplementary Table 1) based on the ITS and D1/D2 sequence analyses.

Among 39 known yeast or yeast-like species isolated from the environment in this study, nine species were frequently isolated, while the other 29 species seem to be rare (Supplementary Table 1). Eighty strains of *Tilletiopsis washingtonensis* were obtained from eight provinces in China, which occupy 32.8% isolate frequency (80 strains/244 total isolated strains). The other frequently isolated species are *Mycosarcoma maydis* (*Ustilago maydis*) (9.4%), *Pseudozyma hubeiensis pro. tem* (6.6%), *Moesziomyces aphidis* (6.1%), *Golubevia pallescens* (5.7%), *Phragmotaenium oryzicola* (5.3%), *Moesziomyces antarcticus* (4.1%), *Gjaerumia minor* (3.7%), and *Meira geulakonigii* (2.9%) (Supplementary Table 1). *T. washingtonensis* commonly occurred on leaves in agreement with the observation of Boekhout (1991, 2011). However, it was also isolated from the soil, rotten wood, and tree bark (Supplementary Table 1). Although *My. maydis* is an important plant pathogen on corn, it was not isolated from other plant materials and soils, but from the skin of cows, sheep (lambs), and shepherds (Supplementary Table 1). The cases of human *My. maydis* infection have been reported (Patel et al., 1995; Teo and Tay, 2006; McNeil and Palazzi, 2012; Peraica et al., 2014; Agata and Marta, 2018). The research from Crotzer and Levetin (1996) indicated that the dispersal of smut spores was intervened by human activity, especially by plant harvesting. The smut spores seem to be transferred from plants to humans or animals by air currents (Crotzer and Levetin, 1996). *Mo. aphidis* was isolated both from plants (leaves), animals (cows), and in the soil. *Ex. reticulatum*, *Go. pallescens*, *Ph. oryzicola*, *Ps. fusiformata*, *Ps. tsukubaensis*, and *T. lilacina* were also isolated from the soil and from plant materials (Supplementary Table 1).

The below analyses illustrate the undescribed diversity of yeasts in *Ustilaginomycotina*, most of which represent rare taxa. A few not included potentially conspecific strains were not available for the study, inactive, or lost. These descriptions were made on a limited number of isolates because more strains could not be obtained despite of extensive sampling and analysis of more than 200 isolates.

The two most frequently used for identification of yeast genetic markers, ribosomal ITS, and D1/D2 domains of LSU proved their utility for identification and delimitation of species in *Ustilaginomycotina*. While nucleotide sequences of D1/D2 domains are often too conservative to distinguish closely related species, this region is useful for phylogenetic analyses. In contrast, the variability of ITS is often sufficient to identify new species from pair-wise similarity comparisons (see below).

### New Taxon Delineation and Phylogenetic Placement

The *Ustilaginomycotina* includes mainly parasitic fungi and few of saprobic yeast or yeast-like members (Begerow et al., 2014). Traditionally, the phenotypic and ecological species concept with species identification based on the combination of host plants and morphological characteristics was applied for the plant pathogenetic fungi (Vánky, 2012; Begerow et al., 2014; Boekhout et al., 2021), but the integrative species concept with the incorporation of phenotypic and ecological characteristics and molecular data (e.g., rDNA and protein genes) have also been used (Stoll et al., 2003, 2005; Begerow et al., 2006, 2014; Sipiczki and Kajdacsi, 2009; McTaggart et al., 2012a,b; Kijpornyongpan and Aime, 2017; Richter et al., 2019; Boekhout et al., 2021). For the yeast or yeast-like fungi, the molecular data, combined with morphological and physiological characters, was mainly used to identify species and diagnose genus (Kurtzman et al., 2011; Begerow et al., 2014, 2017; Richter et al., 2019; Boekhout et al., 2021). Nearly 100 anamorphic yeast or yeast-like species in *Ustilaginomycotina* have been reported (Boekhout et al., 2003; Kurtzman et al., 2011; Begerow et al., 2014; Nasr et al., 2014; Albu et al., 2015; Wang et al., 2015; Sun et al., 2018; Richter et al., 2019), but only few of them have been connected to the sexual taxa or sexual stage, such as *Pseudozyma prolifica* (teleomorph *My. maydis*), *Pseudozyma tsukubaensis* (teleomorph *Macalpinomyces spermophorus*), *Mo. aphidis*, *Mo. antarcticus*, and *Mo. rugulosus* (Wang et al., 2015; Kruse et al., 2017; Tanaka and Honda, 2017; Li et al., 2019; Tanaka et al., 2019). However, most of them were dispersed in separated phylogenetic clades from the teleomorphic genera (Wang et al., 2015). Therefore, the phylogenetic and phenotypic species concept for anamorphic genera was proposed in this study. We tried to compare phylogenetic distances from available data for sexual species and undertaken them as a reference on newly described species in the genera, including both sexual and asexual species.Thus, the phylogenetic species concept was used here for the new asexual species delimitation in the teleomorphic genera because those yeast members have no the host data and sexual stage. This concept also applied in smut and yeasts communities (Kijpornyongpan and Aime, 2017; Richter et al., 2019). The yeast species identification benchmarks suggested by Fell et al. (2000), Scorzetti et al. (2002), Kurtzman and Fell (2006), Kurtzman (2014, 2015), Kurtzman et al. (2015), Vu et al. (2016, 2019), and Li et al. (2020) were also considered, but not followed strictly in this study.

**FIGURE 1:**
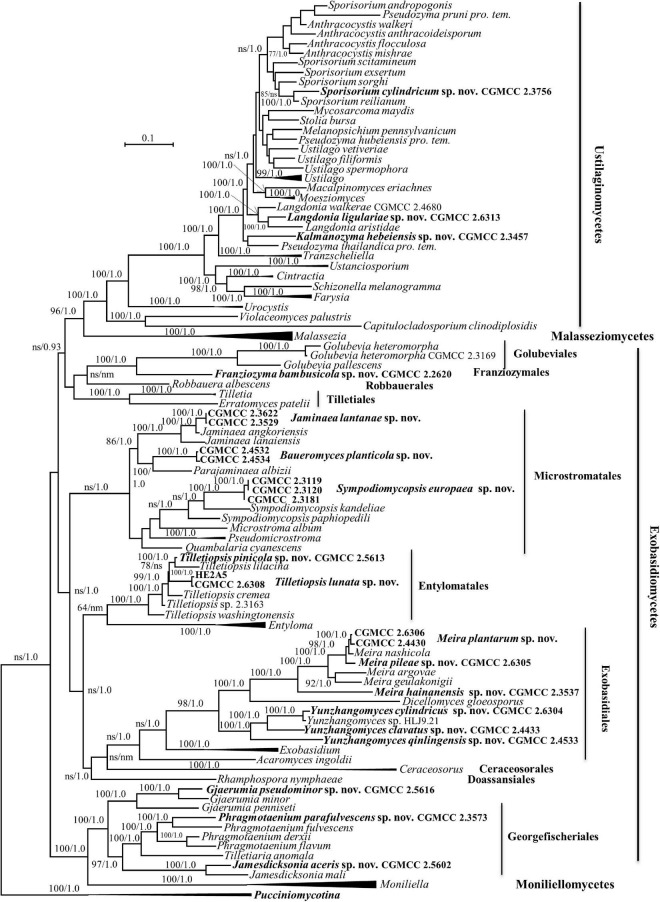
Phylogenetic tree inferred using the combined sequences of SSU rDNA, LSU rDNA D1/D2 domains, Internal Transcribed Spacer (ITS; including 5.8S rDNA), RPB1, RPB2, and TEF1, depicting the phylogenetic positions of new taxa (in bold) within *Ustilaginomycotina*. The tree backbone was constructed using maximum likelihood analysis. Bootstrap percentages of maximum likelihood analysis over 50% from 1,000 bootstrap replicates and posterior probabilities of Bayesian inference above 0.9 are shown respectively from left to right on the deep and major branches. Bar = 0.1 substitutions per nucleotide position. ns, not supported (BP < 50% or PP < 0.9); nm, not monophyletic; the compressed genera are monophyletic, the species in those clades were listed in Table 1 and Supplementary Table S2.

**FIGURE 2:**
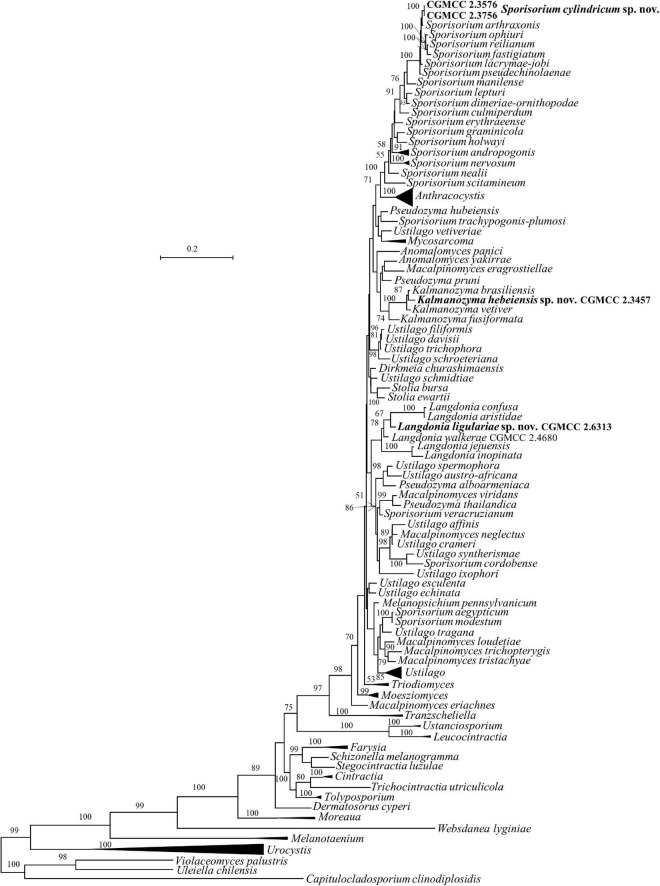
Phylogeny of new yeast or yeast-like species in the *Ustilaginomycetes* inferred from the sequences of the LSU rDNA D1/D2 domains and ITS region (including 5.8S rDNA) by maximum likelihood analysis and over 50% from 1000 bootstrap replicates is shown. Bar = 0.2 substitutions per nucleotide position. the compressed genera are monophyletic, the species in those clades were listed in Table 1 and Supplementary Table S2.

**FIGURE 3:**
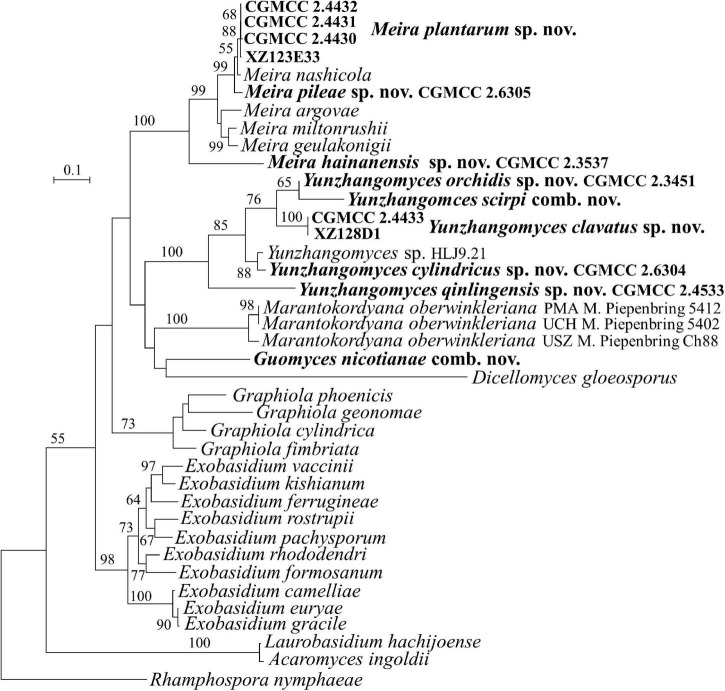
Phylogeny of new yeast or yeast-like species in the *Exobasidiales* inferred from the sequences of the LSU rDNA D1/D2 domains and ITS region (including 5.8S rDNA) by maximum likelihood analysis and over 50% from 1,000 bootstrap replicates is shown. Bar = 0.1 substitutions per nucleotide position.

**FIGURE 4:**
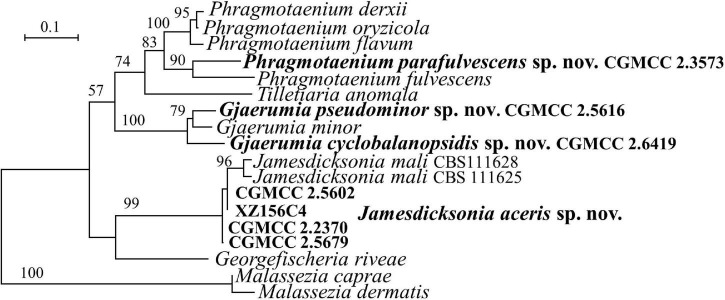
Phylogeny of new yeast or yeast-like species in the *Georgefischeriales* inferred from the sequences of the LSU rDNA D1/D2 domains and ITS region (including 5.8S rDNA) by maximum likelihood analysis and over 50% from 1,000 bootstrap replicates is shown. Bar = 0.1 substitutions per nucleotide position.

**FIGURE 5:**
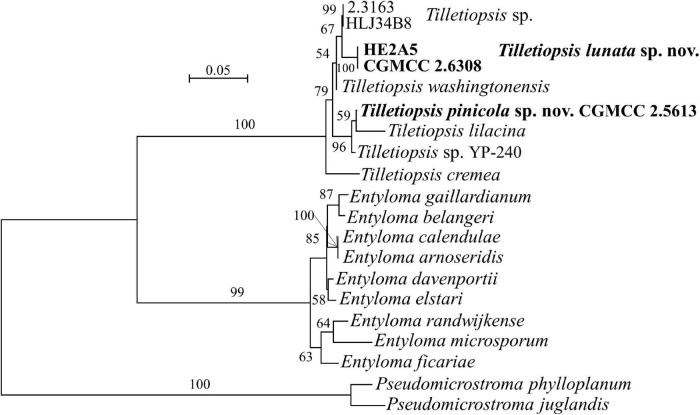
Phylogeny of new yeast or yeast-like species in the *Entylomatales* inferred from the sequences of the LSU rDNA D1/D2 domains and ITS region (including 5.8S rDNA) by maximum likelihood analysis and over 50% from 1,000 bootstrap replicates is shown. Bar = 0.05 substitutions per nucleotide position.

*Note*: Strain CGMCC 2.5613, *Exobasidiomycetes* sp. isolate CK927 (MH483605/MH474509) from lichen biocrust soil in Utah, USA and *Tilletiopsis* sp. isolate YP-240 (KU702544/KU702557) from Duke pine Forest soil in North Carolina, United States have identical D1/D2 sequences, but they differ from each other by 9–10 nt in the ITS region. A multigene approach is needed to determine whether or not they may represent different species.

**FIGURE 6:**
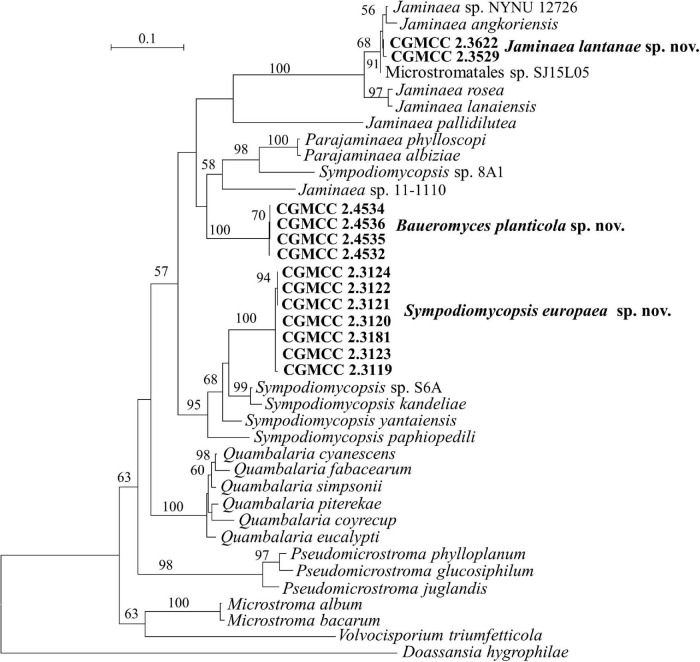
Phylogeny of new yeast or yeast-like species in the *Microstromatales* inferred from the sequences of the LSU rDNA D1/D2 domains and ITS region (including 5.8S rDNA) by maximum likelihood analysis and over 50% from 1,000 bootstrap replicates is shown. Bar = 0.1 substitutions per nucleotide position.

Forty-one strains (Table 1) were grouped into 20 novel species based on the phylogenetic and physiological comparison. Thirty of these strains represent 14 new species that are distributed in the genera *Gjaerumia*, *Jamesdicksonia*, *Jaminaea*, *Kalmanozyma*, *Langdonia*, *Meira*, *Phragmotaenium*, *Sporisorium*, *Sympodiomycopsis*, and *Tilletiopsis*. However, the 11 additional strains, representing six unknown taxa, occur in three unique phylogenetic positions in the phylogenetic trees (Figures 1–6 and Supplementary Figures 1–3) and cannot be assigned to any existing genera. Therefore, three new genera, namely, *Baueromyces* gen. nov., *Franziozyma* gen. nov., and *Yunzhangomyces* gen. nov, are proposed to accommodate these six novel species (see Taxonomy section).

Note that the ex-type strains (or reference strains) of known species were used for sequence similarity analyses for novel species comparisons, and that the GenBank and strain numbers can be found in Supplementary Table 2.

### New Species Identification in the *Ustilaginaceae* (*Ustilaginales*, *Ustilaginomycetes*)

Five strains, CGMCC 2.3576, CGMCC 2.3756, CGMCC 2.3457, CGMCC 2.4680, and CGMCC 2.6313, belong to the family *Ustilaginaceae* (Table 1 and Figures 1, 2).

As taxa within other smut genera, the species concept of *Sporisorium* was traditionally delimited based on the host and morphological characters (Vánky, 2012; Begerow et al., 2014). McTaggart et al. (2012a,b) revised the generic concept of *Sporisorium* based on multi-gene phylogenetic analysis, the morphological characters, and host specificity. In their study, 81 species of *Sporisorium* were used to perform the phylogenetic analysis and 34 species were kept in the revised *Sporisorium* (McTaggart et al., 2012a,b), while other members were transferred to *Anthracocystis*, *Langdonia*, *Stollia*, etc. The asexual species *Pseudozyma graminicola* was transferred to *Sporisorium* based on the phylogenetic analysis (Wang et al., 2015). Our two new isolates, CGMCC 2.3576 and CGMCC 2.3756, were phylogenetically related to *S. arthraxonis*, *S. ophiuri*, *S. fastigiatum*, *S. reilianum*, *S. lacrymae-jobi*, and *S. pseudechinolaenae* and separated from them in the ITS+LSU and multi-gene trees (Figures 1, 2). The six parasitic species differ from each other by 1–6 nt (0.16–1%) and 6–29 nt (0.9–3.9%) in the D1/D2 and the ITS regions, respectively. Strains CGMCC 2.3576 and CGMCC 2.3756 with identical D1/D2 and ITS sequences differ from those six species by more than 22 nucleotide (nt) (3%) mismatches (including substitutions and deletions) and 3–7 nt (0.5–1.1%) in the ITS and the D1/D2 regions, respectively.

The genus *Langdonia* includes ten species, namely, *L. aristidae*, *L. aristidaria*, *L. aristidicola*, *L. clandestina*, *L. confusa*, *L. fraseriana*, *L. goniospora*, *L. inopinata*, *L. jejuensis*, and *L. walkerae* (Begerow et al., 2014; Wang et al., 2015; Alqurashi et al., 2021), among which, nine species have a sexual stage except *L. jejuensis*. Seven of them have rDNA sequences (Supplementary Table 2) and differ from each other by 1–22 nt (0.16–3.6%) and 7–82 nt (1–12%) in the D1/D2 and ITS regions, respectively. Strain CGMCC 2.6313 are placed in the *Langdonia* clade and phylogenetically distinct from other known species (Figures 1, 3). The newly published species *Langdonia walkerae* was described based on sexual characters and molecular data with ten specimens collected from *Aristida stricta* and *Aristida beyrichiana* (*Poaceae*) in the southeastern United States in 2018 (Alqurashi et al., 2021). CGMCC 2.4680, with the asexual yeast stage, was isolated from the leaf of an unidentified plant in China in September 2012. It has two nt differences from *L. walkerae* in the ITS region, which indicated that CGMCC 2.4680 belong to *L. walkerae* (data not shown). This is the first case in the genus *Langdonia* for the connection between the sexual and asexual states. CGMCC 2.6313 has affinity with *L. aristidae* and *L. confusa*, and differs from them by more than 9 nt and 72–75 nt (10%) in the D1/D2 and ITS regions, respectively.

Strain CGMCC 2.3457 locates in the anamorphic genus *Kalmanozyma* clade (Figures 1, 2). It differs from *K. brasiliensis*, *K. vetiver*, and *K. fusiformata* by 5–7 nt in the D1/D2 domain and by 23–71 nt (3–8%) in the ITS region.

### New Taxon Identification in the *Brachybasidiaceae* (*Exobasidiales*, *Exobasidiomycetes*)

The family *Brachybasidiaceae* contains *Brachybasidium*, *Dicellomyces*, *Exobasidiellum*, *Kordyana*, *Meira*, and *Proliferobasidium* (Begerow et al., 2014). Eleven strains representing seven new species are placed in *Brachybasidiaceae* (Table 1, Figures 1, 3 and Supplementary Figures 1, 3). The anamorphic genus *Meira* comprises six species, namely, *Me. argovae*, *Me. geulakonigii*, *Me. miltonrushii*, *Me. nicotianae*, *Me. nashicola*, and *Me. siamensis* (Yasuda et al., 2006; Tanaka et al., 2008; Rush and Aime, 2013; Limtong et al., 2017; Cao et al., 2018). Strains CGMCC 2.4430, CGMCC 2.4431, CGMCC 2.4432, CGMCC 2.6305, CGMCC 2.6306, and CGMCC 2.3537 are clustered in the *Meira* clade (Figures 1, 3 and Supplementary Figures 1, 3). The former five strains form two groups represented by strains CGMCC 2.4430 and CGMCC 2.6305, respectively, and are closely related to *Meira nashicola* (Figures 1, 3). The CGMCC 2.4430 group, including four strains, has identical D1/D2 sequences and differ from one another by 0–7 nt (0–0.9%) in the ITS region, which indicates that they are conspecific. The CGMCC 2.6305 group, represented by a single strain, differs from the CGMCC 2.4430 group by 3 nt in the D1/D2 domain and 42 nt (5.4%) in the ITS region. These two groups differ from *Me. nashicola* by 1–7 nt in the D1/D2 domain, and by 24–27 nt (4–5%) in the ITS region, indicating that they are different species. Strain CGMCC 2.3537 was located in a basal branch in the *Meira* clade, and differs from *Meira* sp. 07F1061 (JX575187) and *Meira* sp. 08F0291 (JX575186) by 6–7 nt and from other know *Meira* species by more than 51 nt (8%) in the D1/D2 domain.

*Meira nicotianae* was described by Cao et al. (2018), which occurred at a basal branch of the *Meira* clade in the LSU and ITS+LSU trees. However, Piepenbring et al. (2020) argued that the genus *Meira* was polyphyletic and that *Me. nicotianae* was separated from the *Meira* clade and was more closely related to *Dicellomyces scirpi* in the ITS + LSU tree. Our analyses (Figure 3 and Supplementary Figure 1) agreed with the result of Piepenbring et al. (2020). The conflicting placement of *Me. nicotianae* might be caused by incomplete taxon sampling. In the analyses of Piepenbring et al. (2020) and our study, more taxa, particularly in *D. scirpi*, were added for the phylogenetic tree construction, which resulted in the separation of *Me. nicotianae* from the genus *Meira*. Therefore, a new genus is proposed for *Me. nicotianae* in the following taxonomy section.

The genus *Dicellomyces* includes four species that parasitize on monocot plants (Begerow et al., 2014), *D. gloeosporus*, the type species, on *Poaceae*, *D. calami* on *Arecaceae*, *D. scirpi* on *Cyperaceae*, and *D. bombacis* on *Bombacaceae*. The latter species has been transferred to the genus *Ceraceosorus* (*Ceraceosoraceae*, *Ceraceosorales*) as *C. bombacis* (Cunningham et al., 1976). Piepenbring et al. (2020) indicated that *D. scirpi* probably represented a new genus based on the ITS + LSU sequence analysis and the comparison of host and morphological characters, including the shape of the sori, the presence of paraphyses, probasidial swellings, and the shape of conidia formed by germinating basidiospores. The molecular analyses from Piepenbring et al. (2020) showed that *D. scirpi* and *D. gloeosporus* were located in different phylogenetic clades, which was in agreement with Nasr et al. (2019) but differs from Tanaka et al. (2008). The phylogenetic incongruence might be caused by insufficient taxon sampling in the study of Tanaka et al. (2008).

Strains CGMCC 2.3451, CGMCC 2.4433, CGMCC 2.4533, CGMCC 2.6304, and XZ128D1 represent four undescribed species (Figures 1, 3) which differ from each other by more than 3% in the D1/D2 domain. They are all closely related to *D. scirpi* but differ from it by 4.4–11.5% in the D1/D2 domain. Our combined ITS, D1/D2 and the combined six-gene phylogenetic analyses confirm that *D. scirpi* and the five new strains are different from *D. gloeosporus* (Figures 1, 3 and Supplementary Figures 1, 3). Therefore, the *D. scirpi* clade was proposed as a new genus in the *Brachybasidiaceae* family (see Taxonomy section).

### New Species Identification in the *Georgefischeriales* (*Exobasidiomycetes*)

Strains CGMCC 2.3573, CGMCC 2.5616, CGMCC 2.6419, CGMCC 2.5679, CGMCC 2.2370, CGMCC 2.5602, and XZ156C4 are placed in *Georgefischeriales* (Figures 1, 4 and Supplementary Figures 1, 2). The genus, *Phragmotaenium*, includes four yeast species and one plant infecting taxon (Begerow et al., 2014; Wang et al., 2015). Strain CGMCC 2.3537 is placed in the *Phragmotaenium* clade and differs from *Ph. indicum*, *Ph. oryzicola*, *Ph. derxii*, *Ph. fulvescens*, and *Ph. flavum* by 6–21 nt (1–3%) in the D1/D2 domain. More than 6% diversity between those taxa was found in the ITS region.

The genus *Gjaerumia* comprises three parasitic smut fungi infecting the *Asparagaceae*, *Melanthiaceae*, and *Xanthorrhoeaceae* plant and two yeast species (Begerow et al., 2014; Wang et al., 2015). *Gjaerumia marneyi*, isolated from the phylloplane of *Hibiscus tiliaceus* (*Malvaceae*), was recently described and known as an asexual culturable yeast based on phylogenetic analysis (Tan et al., 2021). Strains CGMCC 2.5616 isolated from a leaf of an unidentified plant and CGMCC 2.6419 isolated from the leaf of *Cyclobalanopsis* sp. (*Fagaceae*) belong to the *Gjaerumia* clade and differ from the closely related species *G. minor* by 15 (2.5%) and 74 nt (11%) in the D1/D2 and ITS regions, respectively. These two novel strains differ from each other by 8 nt in the D1/D2 domain and 72 nt in the ITS region, which indicates that they belong to different species.

Five species of *Jamesdicksonia*, i.e., *J. dactylidis*, *J. ischaemiana*, *J. irregularis*, and *J. mali*, have available D1/D2 sequence (Supplementary Table 2). They differ from each other by 3–11 nt in this region. Strains CGMCC 2.5679, CGMCC 2.2370, and XZ156C4, isolated from the leaf of an unidentified plant, and CGMCC 2.5602, isolated the leaf of *Acer pectinatum* (*Sapindaceae*), cluster with the *Jamesdicksonia* species and are closely related to the asexual species *J. mali* isolated from apple (*Malus, Rosaceae*) recently described by Richter et al. (2019; Figures 1, 4). The four new strains have 0–3 and 3–5 nt differences in the D1/D2 domain and ITS region, respectively, which indicate that they are conspecific. They differ from *J. mali* CBS 111628 and CBS 111625 by 0–1 nt in the D1/D2 domain. However, there are 27–33 nt (6–7%) differences in the ITS region. They also have more divergence in the assimilation of carbon and nitrogen (see “Taxonomy” section, Table 2). The above phylogenetic and physiological comparisons indicate that they belong to different species. The other species of *Jamesdicksonia* differ from the four new strains by 6–8 nt in the D1/D2 region. *Jamesdicksonia brizae*, without the D1/D2 sequence, differs from the four new strains by more than 53 nt (8%) in the ITS region.

**TABLE 2:** Physiological and biochemical characteristics of new species and their closest relatives.

Characteristic ^a^	Fermentation of glucose	Glucose	Galactose	L-Sorbose	Sucrose	Maltose	Cellobiose	Trehalose	Lactose	Melibiose	Raffinose	Melezitose	Inulin	Solube starch	D-Xylose	L-Arabinose	D-Arabinose	D-Ribose	L-Rhamnose	D-Glucosamine	*N*-Acetyl-D-glucosmine	Methanol	Ethanol	Glycerol	Erythritol	Ribitol	Galactitol	D-Mannitol	D-Glucitol	α-Methyl-D-glucoside	Salicin	D-glueonale	DL-Lactic acid	Succinic acid	Citric acid	Inositol	Hexadecane	Ammonium sulfate	Potassium nitrate	Sodium nitrite	L-lysine	Ethylamine	Cadaverine	Growth in vitamin-free medium	Starch like compounds formation	Growth with 50% glucose	Diazonium Blue B reaction	Hydrolysis of urea	The major ubiquinone	Growth at 17°C	Growth at 25°C	Growth at 30°C	Growth at 37°C
*Sporisorium cylindricum* sp. nov.	-	+	+	–	+	+	+	+	–	+	+	+	–	+	+	–	–	+	+	–	–	–	–	–	–	–	–	–	–	+	+	n	–	+	–	+	–	+	–	–	+	–	–	+	–	–	+	+	n	+	+	n	n
*Kalmanozyma hebeiensis* sp. nov.	–	+	+	–	+	+	+	+	lw	–	w	+	–	–	+	+	w	w	–	+	n	–	+	+	–	+	–	+	+	+	w	n	–	+	+	–	–	+	+	+	w	lw	–	+	–	–	+	+	n	+	+	n	n
*Kalmanozyma vetiver*	–	+	–	l	+	+	w	+	–	–	+	+	–	+	+	–	–	–	–	n	n	–	–	–	–	–	–	l	+	w	w	–	+	+	+	l	n	+	+	+	+	–	w	+	–	+	+	+	10	+	+	+	–
*Kalmanozyma brasiliensis*	–	+	+	+	+	+	+	+	–	–	+	+	+	–	+	+	–	+	–	l	+	–	+	+	+	+	–	+	+	n	+	+	–	+	–	l	–	n	+	+	+	n	l	+	–	–	+	+	n	+	+	+	–
*Langdonia walkerae*	–	+	–	–	+	–	w	w	w	–	w	w	w	w	+	+	–	–	–	–	–	–	–	–	–	–	–	w	–	–	–	n	–	w	–	–	–	+	+	+	+	+	+	–	–	–	+	+	n	+	+	n	n
*Langdonia ligulariae* sp. nov.	–	+	–	–	+	+	+	+	–	–	+	+	w	w	+	+	–	–	–	–	–	–	+	+	–	+	–	+	–	+	–	n	–	–	–	w	–	+	+	w	+	+	+	+	–	–	+	+	n	+	+	n	n
*Langdonia jejuensis*	–	+	l	l	+	+	l	+	+	–	+	+	–	–	+	+	–	+	–	–	l	–	–	–	l	–	–	+	+	–	l	l	–	–	+	–	+	n	+	+	+	–	+	+	–	–	+	+	10	+	+	+	+
*Meira plantarum* sp. nov.	–	+	+	–	+	+	+	+	–	+	+	+	–	–	+	+	–	+	–	–	n	–	–	–	+	–	v	+	+	–	lw	n	–	+, lw	–	–	–	+	+	+	lw, –	+	l	+	–	–	+	+	n	+	+	n	n
*Meira pileae* sp. nov.	–	+	+	–	+	+	+	+	–	+	+	+	+	–	+	+	w	w	–	–	n	–	w	w	w	w	–	w	w	–	w	–	–	lw	–	–	–	+	+	+	+	+	+	+	–	–	+	+	n	+	+	n	n
*Meira nashicola*	–		+				+		–	+		+	–	w		+	+	+					–	–	w	–	–				–	–	–		–	–			+	+	+	+		+	–		+	+	n	+	+	+	n
*Meira hainanensis* sp. nov.	–	+	+	–	+	+	+	+	–	+	+	+	+	+	+	+	+	+	–	–	n	–	+	w	+	lw	+	+	+	–	–	n	–	w	–	–	–	+	w	+	–	–	+	+	–	–	+	+	n	+	+	n	n
*Meira argovae*	–	+	+	–	+	+	+	+	v	v	+	+	+	+	+	+	+	+	–	–	n	–	+	+	+	+	+	+	+	–	+	–	+	+	+	–	n		+	+	n	n	n	v	–	–	+	+	n	+	+	+	–
*Yunzhangomyces orchidis* sp. nov.	–	+	+	+	+	+	+	+	–	l	+	+	+	–	+	+	+	+	–	–	n	–	+	+	+	+	–	+	+	–	+	n	–	lw	lw	–	–	+	+	+	+	+	+	+	–	–	+	+	n	+	+	n	n
*Yunzhangomyces clavatus* sp. nov.	–	+	v	+, lw	+	+	v	+	–	+	+	+	–	–	+	+	–	v	–	–	n	–	+, lw	–	v	v	–	+	+	–	lw, –	n	–	w, –	–	–	–	+	+	+	v	+	+	+	–	–	+	+	n	+	+	n	n
*Yunzhangomyces qinlingensis* sp. nov.	–	+	+	+	+	+	–	+	–	–	+	+	–	–	+	+	lw	+	–	–	–	–	+	–	+	lw	–	+	+	lw	l	n	–	+	+	–	–	+	+	–	lw	lw	lw	+	–	–	+	+	n	+	+	n	n
*Yunzhangomyces cylindricus* sp. nov.	–	+	+	+	+	+	+	+	–	–	+	–	+	–	+	+	+	+	–	–	–	–	+	–	+	+	–	+	+	–	–	n	–	–	–	–	–	+	+	–	+	+	+	+	–	–	+	+	n	+	+	n	n
*Phragmotaenium parafulvescens* sp. nov.	–	+	lw	lw	+	+	–	+	lw	lw	+	+	+	+	+	+	+	–	–	–	–	–	–	+	–	lw	–	+	+	–	–	+	–	+	–	–	–	+	+	–	lw	–	–	+	–	–	+	+	n	+	+	n	n
*Phragmotaenium fulvescens*	–	+	+	v	+	+	+	+	+	+	+	+	+	+	+	+	+	+	–	–	n	–	v	+	+	+	–	+	+	–	–	v	v	+	+	–	n	n	+	+	n	n	n	–	–	–	+	+	10	+	+	+	–
*Gjaerumia pseudominor* sp. nov.	–	+	–	–	+	+	+	+	–	–	–	+	–	+	+	+	–	–	–	–	–	–	–	+	–	–	–	+	+	–	–	n	+	+	+	–	–	+	+	–	+	+	+	w	–	–	+	+	n	+	+	n	n
*Gjaerumia cyclobalanopsidis* sp. nov.	–	+	+	w	+	+	+	+	+	+	+	+	+	–	+	+	+	–	–	–	–	–	–	+	–	w	–	w	w	–	–	n	–	–	–	–	–	+	w	+	w	–	lw	–	–	–	+	+	n	+	+	n	n
*Gjaerumia minor*	–	+	+	v	+	+	+	+	+	+	+	+	+	+	+	+	+	+	–	–	n	–	v	+	v	+	–	+	+	v	–	v	+	+	+	–	n		+	v	n	n	n	–	–	–	+	+	10	+	+	v	–
*Jamesdicksonia aceri* sp. nov.	–	+	v	–	+	+	+	+	v	–	+, w	+, w	v	v	v	+	+	+	+	–	n	–	–	+	+	+	–	+	+	–	–	n	–	v	–	–	–	+	+	–	+	+	+	+	–	–	+	+	n	+	+	n	n
*Jamesdicksonia mali*	–	+	+	–	+	+	+	+	+	+	+	+	+	+	l	+	–	+	w, –	+		–	–	w	w	–	–	+	+	–	–	+	–	+	–	–			+	+	+	w, –	–			–	+	+	n	+	+	w, –	n
*Tilletiopsis pinicola* sp. nov.	–	+	+	–	+	+	–	+	–	w	+	+	–	–	+	+	+	+	–	–	–	–	+	+	+	–	–	–	w	–	–	n	–	w	w	–	–	+	+	–	+	+	+	+	–	–	+	+	n	+	+	n	n
*Tilletiopsis lilacina*	–	+	+	–	+	+	+	+	–	+	+	+	–	+	+	+	+	+	–	–	n	–	–	+	+	+	–	+	+	+	–	+	+	+	+	–	n		+	+	n	n	n	–	–	–	+	+	10	+	+	v	–
*Tilletiopsis lunata* sp. nov.	–	+	+	–	+	+	–	+	–	n	+	+	+	+	+	+	–	+	–	–	–	–	+	+	+	v	–	+	+	–	–	–	–	+	+	–	–	+	+	v	+	–	+	+	–	–	+	+	n	+	+	n	n
*Tilletiopsis washingtonensis*	–	+	v	–	+	+	v	+	–	v	+	+	–	+	+	+	+	+	–	–	n	–	v	+	+	v	–	+	+	v	V	+	+	+	+	–	n	n	+	+	n	n	n	–	–	–	+	+	10	+	+	v	–
*Jaminaea lantanae* sp. nov.	–	+	–	+	l	lw	lw	–	–	–	–	lw	–	–	l	+	–	–	–	l	+	–	+	+	+	–	–	l	lw	lw	lw	–	–	–	–	–	–	+	–	–	+	+	+	+	–	–	+	+	n	+	+	n	n
*Baueromyces planticola* sp. nov.	–	+	+	–	+	+, w	+	+, lw	w	v	+	v	v	–	w	+	–	v	v	–	v	–	w, –	+	+, lw	–	–	+	v	w, –	lw, –	n	–	w, –	–	lw, –	–	+	+, w	–	w	w	w	+	–	–	+	+	n	+	+	n	n
*Sympodiomycopsis europaea* sp. nov.	–	+	+	+	+	+	–	lw	+	+	+	+	–	+, w	+	+, lw	–	+	–	–	n	–	+	+	+	–	–	+	+, lw	+, lw	–	n	–	w, –	lw, –	w, –	–	+	v	–	+	–	+	+	–	–	+	+	n	+	+	n	n
*Sympodiomycopsis kandeliae*	–	+	+, l	+	+	+	+, l	+	+, l	v	+	+	w, –	w, –	+, l	+	+, l	+, l	–	–		–	+, l	+	+	v	w	+	+	+	w	l, w	–	+, w	w	w, –			+, w	v	+, w	+, w	l, w	+	–	+	+	+	n	+	+	+	n
*Sympodiomycopsis paphiopedili*	–	+	+	+	+	+	+	+	+	+	+	+	–	–	+	+	+	+	–	–	n	–	+	+	+	+	–	+	+	+	–	+	–	+	+	+	n		+	+	–	+	–	+	–	+	n	+	10	+	+	+	–
*Franziozyma bambusicola* sp. nov.	–	+	–	–	+	+	+	+	–	–	+	–	+	+	–	–	–	–	–	–		–	–	+	+	–	–	+	+	–	–	n	–	–	–	–	–							w	–	–	+	+	n	+	–	–	–
–																																																					

*^a^+, positive; –, negative; l, latent; w, weak; lw, latent and weak; v, variable; n, not available.*

*Note*: Strain CGMCC 2.6419 differs from the two Japanese strains, NIP003 (AB726595) and NIP007 (AB726598), by 2–3 nt in the D1/D2 domain, which indicate that they may be conspecific.

### New Species Identification in the *Entylomatales* (*Exobasidiomycetes*)

Three species, namely, *Tilletiopsis cremea*, *T. lilacina*, and *T. washingtonensis*, were included in the revised genus *Tilletiopsis* (Wang et al., 2015). Strains HE6AB1, HE2A5, and CGMCC 2.5613 are clustered in the *Tilletiopsis* clade (Figures 1, 5 and Supplementary Figures 1, 3). The former two strains have 2 nt differences in both the D1/D2 and ITS regions, indicating their conspecificity. These two strains differ from strain CGMCC 2.5613 by 12 nt in the D1/D2 domain and by 23–24 nt in the ITS region. Strains HE6AB1 and HE2A5 are closely related to *T. washingtonensis* and differ from it by 3 and 10–12 nt (∼2%) in the D1/D2 domain and ITS region, respectively. Strain CGMCC 2.5613 has identical D1/D2 sequences with *Tilletiopsis lilacina*. However, they differ from each other by 11 nt (∼2%) in the ITS region. Physiological profiles of HE6AB1, HE2A5, and CGMCC 2.5613 differed from their closely related species *T. washingtonensis* and *T. lilacina* (see “Taxonomy” section, Table 2), and the phylogenetic analysis both indicated that they belong to two new species in *Tilletiopsis*.

### New Species Identification in the *Microstromatales* (*Exobasidiomycetes*)

The *Microstromales* comprises *Jaminaea*, *Parajaminaea*, *Pseudomicrostroma*, *Microstroma*, *Quambalaria*, *Sympodiomycopsis*, and *Volvocisporium*. *Parajaminaea*, *Pseudomicrostroma*, and *Microstroma* are teleomorphic genera and contain both sexual and asexual species. The other genera in *Microstromales* belong to strictly anamorphic fungi. Our 14 isolates (Table 1) in *Microstromales* were all placed outside the known sexual genera (Figures 1, 6 and Supplementary Figures 1, 3).

Sipiczki and Kajdacsi (2009) proposed the genus *Jaminaea* based on rDNA phylogenetic analysis. The genus currently comprises four species, namely, *J. angkorensis*, *J. lanaiensis*, *J. pallidilutea*, and *J. rosea* (Sipiczki and Kajdacsi, 2009; Kijpornyongpan and Aime, 2017; Nasr et al., 2017). Strains CGMCC 2.3529, CGMCC 2.3622, and CGMCC 3662 have identical ITS and D1/D2 sequences. They are closely related to *Jaminaea angkorensis* (Figures 1, 6) and differ from it by 3 and 19 nt (3%) in the D1/D2 and ITS region, respectively.

Three species, namely, *Sympodiomycopsis paphiopedili*, *S. kandeliae*, and *S. yantaiensis*, were placed in the genus *Sympodiomycopsis* (Sugiyama et al., 1991; Wei et al., 2011; Chen et al., 2013). Strains CGMCC 2.3119, CGMCC 2.3120, CGMCC 2.3121, CGMCC 2.3122, CGMCC 2.3123, CGMCC 2.3124, and CGMCC 2.3181 differ from one another by 2 nt in the D1/D2 domain and by 3 nt in the ITS region, indicating that they are the same species. They differ from *Sympodiomycopsis yantaiensis*, *S. paphiopedili*, and *S. kandeliae* by 19–23 nt (4%) in the D1/D2 domain and by 60 nt (9%) in the ITS region.

Strains CGMCC 2.4532, CGMCC 2.4534, CGMCC 2.4535, and CGMCC 2.4536 form a separate branch with 100% BP support and are closely related to the genera *Parajaminaea* and *Jaminaea* in the *Microstromatales* (Figures 1, 6). These four strains have identical ITS sequences and 1 nt difference in the D1/D2 domain, indicating that they are conspecific. The D1/D2 and ITS sequence blast results showed that the four strains differ from the known species of *Jaminaea*, *Parajaminaea*, *Pseudomicrostroma*, *Microstroma*, *Quambalaria*, *Sympodiomycopsis*, and *Volvocisporium* by more than 20 nt (3%) and 90 nt (13%), respectively. The above data indicate that strains CGMCC 2.4532, CGMCC 2.4534, CGMCC 2.4535, and CGMCC 2.4536 represent a new genus in the *Microstromatales* because they cannot be placed in the existing genera in *Microstromatales*.

*Note*: The CGMCC 2.4532 group has identical ITS sequences with two strains 5CL1 (KJ460375) and 4FL2 (KJ460376) from Brazil, and identical D1/D2 sequences with strain BMA 85 (MH908976) from Brazil, which indicates they are conspecific.

### New Species Identification in the *Exobasidiomycetes* Without Affiliation to a Known Order

Two strains, XZ4C4 and XZ4A1, have the same sequences in both the ITS and D1/D2 regions. A BLASTn search using the D1/D2 sequence of XZ4C4 showed that the top matched sequences were that of species in *Microstromales*, such as *Ps. phylloplanum* and *Quambalaria cyanescens*, with 94% similarity. However, the best match is with *Golubevia pallescens* and ‘*Entyloma dahliae*’ with 57–79% coverage and 79–83% similarity using ITS sequences as the query. To confirm the phylogenetic position of these two strains, a multiple gene phylogenetic tree was constructed (Figure 1). Strain XZ4C4, *G. pallescens*, and *Golubevia heteromorpha*, which was recently reported by Richter et al. (2019), form a clade with strong support (92% BP and 1.0 PP), but the former is clearly different from the latter since they are located in separate branches (Figure 1 and Supplementary Figures 1, 3). The sequence similarities in the D1/D2 and ITS regions between strain XZ4C4 and *G. pallescens* are 90.8 (535/589) and 71% (449/632), respectively, which are too distant to place XZ4C4 in the genus *Golubevia* and order *Golubeviales*. The above analyses indicated that strain XZ4C4 could represent a new order, distinct from *Golubeviales*. Therefore, *Franziozyma bambusoicola* gen. et sp. nov., *Franziozymaceae* fam. nov., and *Franziozymales* ord. nov. are proposed for strains XZ4C4 and XZ4A1.

### Taxonomy

#### New Taxa in *Ustilaginaceae (Ustilaginales, Ustilaginomycetes)*

##### *Sporisorium cylindricum* Q.M. Wang, Y.Y. Li, M. Groenew. and M.M. Wang sp. nov. — MycoBank 839570

*Etymology*: the specific epithet *cylindricum* refers to the cell morphology of the type strain.

**FIGURE 7:**
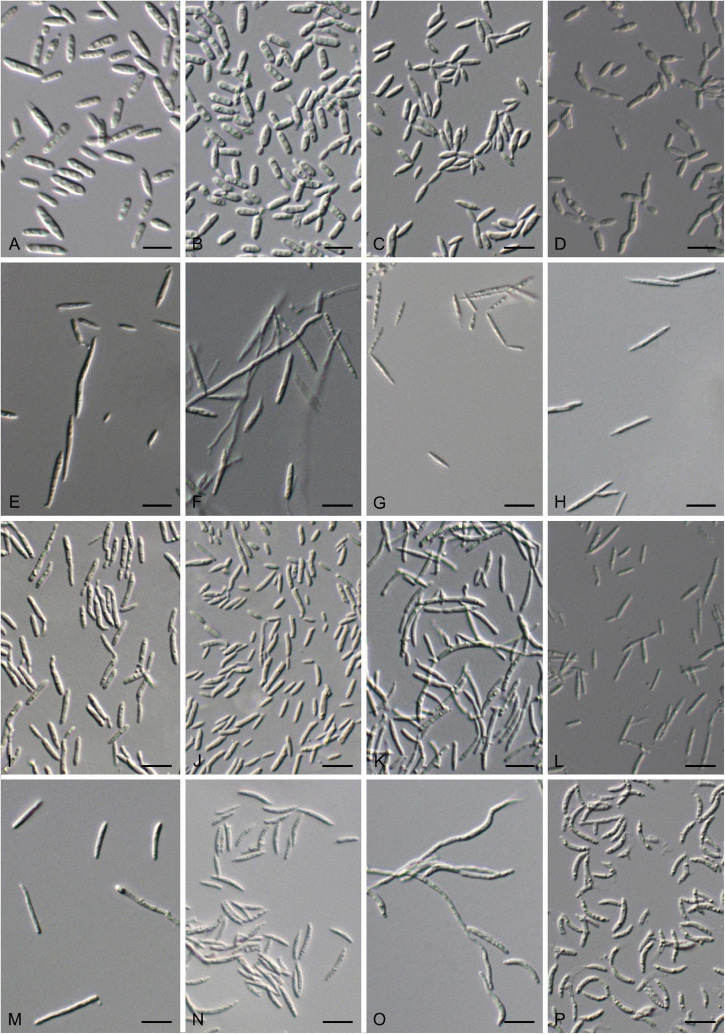
Vegetative cells grown in Yeast Mold (YM) broth for 7 days at 17°C and ballistoconidia produced on corn meal agar after 7 days at 17°C. **(A)** *S. cylindricum* CGMCC 2. 3756^T^; **(B)** *K. hebeiensis* CGMCC 2.3457^T^; **(C)** *L. walkerae* CGMCC 2. 4680; **(D)** *L. ligulariae* CGMCC 2. 6313; **(E)** *M. plantarum* CGMCC2.4430^T^; **(F,G)** *M. pileae* CGMCC 2.6305^T^; **(H)** *M. hainanensis* CGMCC 2.3537^T^; **(I)** *Y. orchidis* CGMCC 2.3451^T^; **(J)** *Y. clavatus* CGMCC 2.4433^T^; **(K)** *Y. qinlingensis* CGMCC 2.4533^T^; **(L)** *Y. cylindricus* CGMCC 2.6304^T^; **(M,N)** *P. parafulvescens* CGMCC 2.3573^T^; **(O,P)** *G. pseudominor* CGMCC 2.5616^T^. Bars = 10 μm. **(G,N,P)** are ballistoconidia.

After 7 days at 17°C in YM broth, cells are cylindrical, 1.5–3.5 × 6.5–14.0 μm and single, a sediment is produced, budding is polar (Figure 7A). After 1 month at 17°C, a sediment and a film are formed. The streak culture is smooth, dull, whitish-cream, butyrous, and has an entire margin after 1 month at 17°C on YM agar. Pseudohyphae are absent in Dalmau plate culture on corn meal agar. Sexual structures are not produced on Potato Dextrose Agar (PDA), Yeast Mold (YM), CM, and V8 agars. Ballistoconidia are not observed.

Glucose is not fermented. Glucose, galactose, sucrose, maltose, cellobiose, trehalose, melibiose, raffinose, melezitose, soluble starch, D-ribose, D-xylose, L-rhamnose, α-Methyl-D-glucoside, salicin, succinic acid, and inositol are assimilated. L-sorbose, lactose, inulin, D-arabinose, L-arabinose, D-glucosamine, *N*-Acetyl-D-glucosmine, ethanol, glycerol, D-mannitol, D-glucitol, methanol, erythritol, ribitol, galactitol, D-glueonale, DL-lactic acid, citric acid, and hexadecane are not assimilated. Ammonium sulfate and L-lysine are assimilated. Potassium nitrate, sodium nitrite, ethylamine, and cadaverine are not assimilated. Maximum growth temperature is 30°C. Growth does occur in a vitamin-free medium. No starch-like substrate is produced. Growth does not occur on 50% (w/w) glucose-yeast extract agar. Urease and Diazonium blue B reactions are positive.

*Typus*: China, Tibet, obtained from a leaf of an unidentified plant, Oct. 2007, Qi-Ming Wang, holotype CGMCC 2. 3756^T^ preserved in a metabolically inactive state in the China General Microbiological Culture Collection Center (CGMCC), Beijing, China. Ex-type CBS 15755 is deposited at the CBS collection of the Westerdijk Fungal Biodiversity Institute, Utrecht, Netherlands. Kunming county, Yunnan province, obtained from a leaf of an unidentified plant, May. 2007, Qi-Ming Wang, paratype CGMCC 2.3576.

*Note*: *S. arthraxonis*, *S. ophiuri*, *S. fastigiatum*, *S. reilianum*, *S. lacrymae-jobi*, and *S. pseudechinolaenae* are all parasitized on *Poaceae*. The two yeasts, CGMCC 2.3576 and CGMCC 2.3756, also isolated from leaves of the plant. Unfortunately, those plants were not identified. Except the worldwide distributed *S. reilianum*, the above five parasitic species mostly occur in the tropic region including southern China and Southeast Asia (Vánky, 2012). The two strains of *S. cylindricum*, CGMCC 2.3576 and CGMCC 2.3756, were isolated from the plant leaf collected in Yunan province (southern China) and Hanan province (southern China), respectively, which indicated that they have similar ecological and biogeographical characters to those parasitic species.

##### *Kalmanozyma hebeiensis* Q.M. Wang, Y.Y. Li, M. Groenew. and M.M. Wang sp. nov. — MycoBank 839571

*Etymology*: the specific epithet *hebeiensis* refers to the geography from which the type strain was isolated.

After 7 days at 17°C in YM broth, cells are cylindrical, 1.7–3.0 × 5.8–10.0 μm, and single or in pairs, a sediment is produced, budding is polar (Figure 7B). After 1 month at 17°C, a sediment and an incomplete ring are produced. The streak culture is smooth, gloomy, yellowish-cream, butyrous, and has an entire margin after 1 month at 17°C on YM agar. Pseudohyphae are formed in Dalmau plate culture on cornmeal agar. Sexual structures are not produced on PDA, YM, CM, and V8 agars. Ballistoconidia are not observed.

Glucose is not fermented. Glucose, galactose, sucrose, maltose, cellobiose, trehalose, lactose (latent and weak), raffinose (weak), melezitose, D-xylose, L-arabinose, D-arabinose (weak), D-ribose (weak), D-glucosamine, ethanol, glycerol, ribitol, D-mannitol, D-glucitol, α-Methyl-D-glucoside, salicin (weak), succinic acid and citric acid are assimilated. L-sorbose, melibiose, inulin, soluble starch, L-rhamnose, methanol, erythritol, galactitol, DL-lactic acid, inositol, and hexadecane are not assimilated. Ammonium sulfate, potassium nitrate, sodium nitrite, L-lysine (weak), and ethylamine (latent and weak) are assimilated. Cadaverine is not assimilated. Optimal growth is at 17–25°C. Growth does occur in a vitamin-free medium. No starch-like substrate is produced. Growth does not occur on 50% (w/w) glucose-yeast extract agar. Urease and Diazonium blue B reactions are positive.

Physiologically, *K. hebeiensis* differs from its closely related species, *K. brasiliensis* and *K. vetiver*, in its inability to assimilate L-sorbose and inositol (Table 2).

*Typus*: China, Hebei province, obtained from a leaf of an unidentified plant, October 2007, Qi-Ming Wang, holotype CGMCC 2.3457^T^ preserved in a metabolically inactive state in the CGMCC, Beijing, China. Ex-type CBS 15483 is deposited at the CBS collection of the Westerdijk Fungal Biodiversity Institute, Utrecht, Netherlands.

*Note*: *K. brasiliensis* was isolated from the intestinal tract of chrysomelid larva associated with roots of *Saccharum* in Brazil (Oliveira et al., 2014). *K. fusiformata* was obtained from a barley leaf and from cauliflower (Boekhout, 2011). *K. vetiveriae* was isolated from phylloplane of *Vetiveria zizanioides* in Thailand. CGMCC 2.3457 was isolated from the plant. The host-substratum data indicated that species of *Kalmanozyma* are associated with the plant.

##### Yeast Stage Description of ***Langdonia walkerae***

*Langdonia walkerae* was described by Alqurashi et al. (2021) based on a sexual stage. A yeast strain, CGMCC 2. 4680, isolated from a leaf of an unidentified plant, collected in September 2012 in China has 99.6% sequence similarity to *L. walkerae* in the ITS region, which indicated they belong to the same species. Here we described the yeast morphological and physiological characters as the asexual state of *L. walkerae.*

After 7 days at 17°C in YM broth, cells are fusiformis, long ovoid, and cylindrical, 1.1–2.5 × 3.5–11.8 μm and single, a sediment is produced, and budding is polar (Figure 7C). After 1 month at 17°C, a sediment and a film are produced. The streak culture is pale, yellowish-brown, flat, butyrous, glossy, slightly granulate, and has an entire margin after 1 month at 17°C on YM agar. Pseudohyphae are formed in Dalmau plate culture on cornmeal agar. Sexual structures are not produced on PDA, YM, CM, and V8 agars. Ballistoconidia are not observed.

Glucose is not fermented. Glucose, sucrose, cellobiose (weak), trehalose (weak), lactose (weak), raffinose (weak), melezitose (weak), inulin (weak), soluble starch (weak), D-xylose, L-arabinose, D-mannitol (weak), and succinic acid (weak) are assimilated. Galactose, L-sorbose, maltose, melibiose, D-arabinose, D-ribose, L-rhamnose, D-glucosamine, *N*-Acetyl-D-glucosmine, methanol, ethanol, glycerol, erythritol, ribitol, galactitol, D-glucitol, α-Methyl-D-glucoside, salicin, DL-lactic acid, citric acid, myo-inositol, and hexadecane are not assimilated. Ammonium sulfate, potassium nitrate, sodium nitrite, L-lysine, ethylamine, and cadaverine are assimilated. Optimal growth is at 17–25°C. Growth does not occur in vitamin-free medium. No starch-like substrate is produced. Growth does not occur on 50% (w/w) glucose-yeast extract agar Urease and Diazonium blue B reactions are positive.

Physiologically, *L. walkerae* differs from its closely related species, *L. jejuensis*, and *L. ligulariae*, in its inability to assimilate maltose and its ability to assimilate succinic acid (Table 2).

##### *Langdonia ligulariae* Q.M. Wang, Y.Y. Li, M. Groenew. and M.M. Wang sp. nov. — MycoBank 839578

*Etymology*: the specific epithet, *ligulariae*, refers to *Ligularia*, the plant genus from which the type strain was isolated.

After 7 days at 17°C in YM broth, cells are cylindrical to elongate, 1.5–2.7 × 4.0–12.0 μm and single, a sediment is produced, and budding is polar (Figure 7D). After 1 month at 17°C, a sediment and a ring are produced. The streak culture is yellowish-brown, flat, butyrous, arachnoid, and has an entire margin after 1 month at 17°C on YM agar. Pseudohyphae or hyphae are formed in Dalmau plate culture on cornmeal agar. Sexual structures are not produced on PDA, YM, CM, and V8 agars. Ballistoconidia are not observed.

Glucose is not fermented. Glucose, sucrose, maltose, cellobiose, trehalose, raffinose, melezitose, inulin (weak), soluble starch (weak), D-xylose, L-arabinose, ethanol, glycerol, ribitol, D-mannitol, α-Methyl-D-glucoside, and inositol (weak) are assimilated. Galactose, L-sorbose, lactose, melibiose, D-arabinose, D-ribose, L-rhamnose, D-glucosamine, *N*-Acetyl-D-glucosmine, methanol, erythritol, galactitol, D-glucitol, salicin, DL-lactic acid, succinic acid, citric acid, and hexadecane are not assimilated. Ammonium sulfate, potassium nitrate, sodium nitrite (weak), L-lysine, ethylamine, and cadaverine are assimilated. Optimal growth is at 17–25°C. Growth does occur in the vitamin-free medium. No starch-like substrate is produced. Growth does not occur on 50% (w/w) glucose-yeast extract agar Urease and Diazonium blue B reactions are positive.

Physiologically, *L. ligulariae* differs from its closely related species, *L. walkerae* and *L. jejuensis*, in its inability to assimilate lactose and its ability to use ethanol, glycerol, ribitol, and α-Methyl-D-glucoside (Table 2).

*Typus*: China, Tibet, obtained from a leaf of *Ligularia tsangchanensis*, September 2012, Qi-Ming Wang, holotype CGMCC 2. 6313^T^ preserved in a metabolically inactive state in the CGMCC, Beijing, China. Ex-type CBS 15581 is deposited at the CBS collection of the Westerdijk Fungal Biodiversity Institute, Utrecht, Netherlands.

*Note*: All sexual *Langdonia* species infected the plant of *Aristida* (*Poaceae*). The asexual yeast species *L. jejuensis* was isolated from a leaf of *Citrus unshiu* (*Rutaceae*) in South Korea. CGMCC 2.6313 was isolated from a leaf of *Ligularia tsangchanensis* (*Asteraceae*) in Tibet, China. Although CGMCC 2.6313 and *L. jejuensis* were all isolated from the leaf of plant (*Asteraceae* and *Rutaceae*), they differ from the parasitic species of *Langdonia* whose host is the *Poaceae* grass, which indicated that the asexual yeast stage and the sexual stage of *Langdonia* may have different ecological inches in nature.

#### Brachybasidiaceae (Exobasidiales, Exobasidiomycetes)

##### *Meira plantarum* Q.M. Wang, Y.Y. Li, M. Groenew. and M.M. Wang sp. nov. — MycoBank 839580

*Etymology*: the specific epithet *plantarum* refers to the substrates from which the type strain was isolated.

After 7 days at 17°C in YM broth, cells are fusiform and cylindrical to elongate, 1.7–2.1 × 4.2–28.3 μm and single, a sediment is produced, and budding is polar (Figure 7E). After 1 month at 17°C, a sediment a film and are produced. On YM agar, after 1 month at 17°C, the colonies are firm to tough, whitish at first before becoming pale yellowish-brown with a velvety to the pruinose surface, and the margin is eroded. Hyphae are formed in Dalmau plate culture on cornmeal agar. Sexual structures are not produced on PDA, YM, CM, and V8 agars. Ballistoconidia are not observed.

Glucose is not fermented. Glucose, galactose, sucrose, maltose, cellobiose, trehalose, melibiose, raffinose, melezitose, D-xylose, L-arabinose, D-ribose, erythritol, galactitol (variable), D-mannitol, D-glucitol, salicin (latent and weak), and succinic acid (latent and weak) are assimilated. L-sorbose, lactose, inulin, soluble starch, D-arabinose, L-rhamnose, D-glucosamine, methanol, ethanol, glycerol, ribitol, α-Methyl-D-glucoside, DL-lactic acid, citric acid, inositol, and hexadecane are not assimilated. Ammonium sulfate, potassium nitrate, sodium nitrite, ethylamine, and cadaverine (latent) are assimilated. L-lysine (or latent and weak) is not assimilated. Optimal growth is at 17–25°C. Growth does occur in the vitamin-free medium. No starch-like substrate is produced. Growth does not occur on 50% (w/w) glucose-yeast extract agar Urease and Diazonium blue B reactions are positive.

Physiologically, *M. plantarum* differs from its closely related species, *M. nashicola* and *M. pileae*, in its inability to assimilate D-arabinose (Table 2).

*Typus*: China, Fuzhou county, Fujian province, obtained from a leaf of an unidentified plant, Oct. 2011, Qi-Ming Wang, holotype CGMCC 2.4430^T^ preserved in a metabolically inactive state in the CGMCC, Beijing, China. Ex-type CBS 12491 is deposited at the CBS collection of the Westerdijk Fungal Biodiversity Institute, Utrecht, Netherlands. Fuzhou county, Fujian province, obtained from a leaf of an unidentified plant, October 2011, Qi-Ming Wang, paratypes CGMCC 2.4431 and CGMCC 2.4432. Beibengxiang, Motuo county, Tibet, obtained from a leaf of an unidentified plant, September 2014, Qi-Ming Wang, paratype CGMCC 2.6306.

##### *Meira pileae* Q.M. Wang, Y.Y. Li, M. Groenew. and M.M. Wang sp. nov. — MycoBank 839582

*Etymology*: the specific epithet *pileae* refers to *Pilea*, the plant genus from which the type strain was isolated.

After 7 days at 17°C in YM broth, cells are cylindrical to elongate, 1.3–2.3 × 5.0–25.0 μm and single, a sediment is produced, budding is polar, hyphae are narrow, and 1.2–2.5 μm (Figure 7F). After 1 month at 17°C, a film and a sediment are produced. On YM agar, after 1 month at 17°C, the colonies are firm to tough with a whitish velvety to the pruinose surface, dull, and the margin is eroded. Hyphae are formed in Dalmau plate culture on cornmeal agar. Sexual structures are not produced on PDA, YM, CM, and V8 agars. Ballistoconidia are fusiform or cylindrical (1.0–2.1 × 6.7–12.5 μm; Figure 7G).

Glucose is not fermented. Glucose, galactose, sucrose, maltose, cellobiose, trehalose, melibiose, inulin, raffinose, melezitose, D-xylose, L-arabinose, D-arabinose (weak), ethanol (weak), glycerol (weak), ribose (weak), erythritol (weak), D-mannitol (weak), D-glucitol (weak), salicin, and succinic acid (latent and weak) are assimilated. L-sorbose, lactose, galactitol, soluble starch, L-rhamnose, D-glucosamine, methanol, α-Methyl-D-glucoside, D-glueonale, DL-lactic acid, citric acid, inositol, and hexadecane are not assimilated. Ammonium sulfate, potassium nitrate, sodium nitrite, L-lysine, ethylamine, and cadaverine are assimilated. Optimal growth is at 17–25°C. Growth does occur in the vitamin-free medium. No starch-like substrate is produced. Growth does not occur on 50% (w/w) glucose-yeast extract agar Urease and Diazonium blue B reactions are positive.

Physiologically, *Me. pileae* differs from its closely related species, *Me. nashicola* and *Me. Plantarum*, in its ability to assimilate inulin (Table 2).

*Typus*: China, Beibengxiang, Motuo county, Tibet, obtained from a leaf of *Pilea* sp., September 2014, Qi-Ming Wang, holotype CGMCC 2.6305^T^ preserved in a metabolically inactive state in the CGMCC, Beijing, China. Ex-type CBS 144915 is deposited at the CBS collection of the Westerdijk Fungal Biodiversity Institute, Utrecht, Netherlands.

##### *Meira hainanensis* Q.M. Wang, Y.Y. Li, M. Groenew. and M.M. Wang sp. nov. — MycoBank 839584

*Etymology*: the specific epithet *hainanensis* refers to the geographic origin of the type strain, Hainan province, China.

After 7 days at 17°C in YM broth, cells are fusiform and cylindrical to elongate, 1.0–1.7 × 6.7–20.0 μm and single, a sediment is produced, budding is polar, hyphae are narrow (1.2–2.5 μm; Figure 7H). After 1 month at 17°C, a thick film and a sediment are produced. The colonies are firm to tough, whitish at first, before becoming pale yellowish-brown with the farinose surface, and the margin is eroded after 1 month at 17°C on YM agar. Hyphae are formed in Dalmau plate culture on cornmeal agar. Sexual structures are not produced on PDA, YM, CM, and V8 agars. Ballistoconidia are not observed.

Glucose is not fermented. Glucose, galactose, sucrose, maltose, cellobiose, trehalose, melibiose, raffinose, melezitose, inulin, soluble starch, D-xylose, L-arabinose, D-arabinose, D-ribose, ethanol, glycerol (weak), erythritol, ribitol (latent and weak), galactitol, D-mannitol, D-glucitol, and succinic acid (weak) are assimilated. L-sorbose, lactose, L-rhamnose, D-glucosamine, methanol, α-Methyl-D-glucoside, salicin, DL-lactic acid, citric acid, inositol, and hexadecane are not assimilated. Ammonium sulfate, potassium nitrate (weak), sodium nitrite, and cadaverine are assimilated. L-lysine and ethylamine are not assimilated. Optimal growth is at 17–25°C. Growth does occur in the vitamin-free medium. No starch-like substrate is produced. Growth does not occur on 50% (w/w) glucose-yeast extract agar Urease and Diazonium blue B reactions are positive.

Physiologically, *Me. hainanensis* differs from its closely related species *Me. argovae* in its inability to assimilate salicin and citric acid (Table 2).

*Typus*: China, Wuzhishan Montain, Hainan province, obtained from a leaf of an unidentified plant, May 2007, Qi-Ming Wang, holotype CGMCC 2.3537^T^ preserved in a metabolically inactive state in the CGMCC, Beijing, China. Ex-type CBS 15497 is deposited at the CBS collection of the Westerdijk Fungal Biodiversity Institute, Utrecht, Netherlands.

*Note*: Limtong et al. (2017) indicated that species of *Meira* seem to be relate to plants and organisms associated with plants. *Me. argovae* and *Me. geulakonigii* were isolated from mite cadavers (*Tetranychus cinnabarinus* and *Phyllocoptruta oleivira*) from citrus leaves (*Ricinus communis* and *Citrus paradisi*) in Israel (Boekhout et al., 2003). *Me. nashicola*, *Me. miltonrushii*, and *Me. siamensis* were obtained from the leaf or fruit of plant (Yasuda et al., 2006; Tanaka et al., 2008; Rush and Aime, 2013; Limtong et al., 2017). Our three described *Meira* species were all isolated from the leaf of plant (Table 1). Some strains of *Meira* had been reported as endophytes of plant species (Paz et al., 2007a; Rush and Aime, 2013). *Me. argovae*, *Me. geulakonigii*, and *Me. nashicola* were proposed to use as a biological control agent against mites and powdery mildew fungi (Boekhout et al., 2003; Sztejnberg et al., 2004; Paz et al., 2007b; Gerson et al., 2008). The biological control property of our described *Meira* species, *Me. miltonrushii* and *Me. siamensis* need to be tested in the future.

##### *Yunzhangomyces* Q.M. Wang, E. Tanaka, M. Groenew., and D. Begerow *gen.* nov. — MycoBank 839581

*Etymology*: the genus is named in honor of Yun-Zhang Wang for his pioneering work on the taxonomy of smuts.

This genus is proposed for the branch represented by *Dicellomyces scirpi*, which formed a separate branch from the genera in the *Brachybasidiaceae* family (*Exobasidiales*, *Exobasidiomycetes*). The genus is mainly circumscribed by the description of *Dicellomyces scirpi* and the phylogenetic analysis of the six-genes sequences (Figure 1).

This genus includes sexual and asexual species. Sexual member infecting *Scirpus sylvaticus* (*Cyperaceae*); basidia developing in gelatinous basidiocarps breaking through epidermis, swollen, not persistent probasidia, with paraphyses, sterigmata 2; producing allantoid or coiled conidia (Parmasto, 1968; Reid, 1976; Ingold, 1985; Piepenbring et al., 2020). Asexual species present butyrous, yellow or brown colonies, smooth or eroded margin with budding cells present.

*Type species*: *Yunzhangomyces scirpi* (Raitv.) Q.M. Wang, E. Tanaka, M. Groenew. and D. Begerow.

##### *Yunzhangomyces scirpi* (Raitv.) Q.M. Wang, E. Tanaka, M. Groenew. and D. Begerow, *comb.* nov. — MycoBank 839585

*Basionym*: *Dicellomyces scirpi* Raitv., in Parmasto, Eesti NSV Tead. Akad. Toim. 17(2): 223 (1968).

##### *Yunzhangomyces orchidis* Q.M. Wang, E. Tanaka, M. Groenew. and D. Begerow sp. nov. — MycoBank 839587

*Etymology*: the specific epithet *orchidis* refers to plant host, *Orchidaceae* sp., from which the type strain was isolated.

After 7 days at 17°C in YM broth, cells are cylindrical to elongate, .08–1.7 × 2.8–16.7 μm and single, a sediment is produced, budding is polar, and hyphae are narrow (1.2-2.5 μm; Figure 7I). After 1 month at 17°C, a thick film and a sediment are produced. The streak culture is pale yellowish to brown with smooth and glistening surface, butyrous, and has an entire margin after 1 month at 17°C on YM agar. Pseudohyphae are formed in Dalmau plate culture on cornmeal agar. Sexual structures are not produced on PDA, YM, CM, and V8 agars. Ballistoconidia are not observed.

Glucose is not fermented. Glucose, galactose, L-sorbose, sucrose, maltose, cellobiose, trehalose, melibiose (latent), raffinose, melezitose, inulin, D-xylose, L-arabinose, D-arabinose, D-ribose, ethanol, glycerol, erythritol, ribitol, D-mannitol, D-glucitol, salicin, succinic acid (latent and weak), and citric acid (latent and weak) are assimilated. Lactose, soluble starch, L-rhamnose, D-glucosamine, methanol, galactitol, α-Methyl-D-glucoside, DL-lactic acid, inositol, and hexadecane are not assimilated. Ammonium sulfate, potassium nitrate, sodium nitrite, L-lysine, ethylamine, and cadaverine are assimilated Optimal growth is at 17–25°C. Growth does occur in the vitamin-free medium. No starch-like substrate is produced. Growth does not occur on 50% (w/w) glucose-yeast extract agar Urease and Diazonium blue B reactions are positive.

Physiologically, *Y. orchidis* differs from its closely related culturable species *Y. clavatus* in its ability to assimilate inulin, D-arabinose, and glycerol (Table 2).

*Typus*: China, Wuzhishan Montain, Hainan province, obtained from a leaf of *Orchidaceae* sp., Apr. 2007, Qi-Ming Wang, holotype CGMCC 2.3451^T^ preserved in a metabolically inactive state in the CGMCC, Beijing, China. Ex-type CBS 15753 is deposited at the CBS collection of the Westerdijk Fungal Biodiversity Institute, Utrecht, Netherlands.

##### *Yunzhangomyces clavatus* Q.M. Wang, E. Tanaka, M. Groenew. and D. Begerow sp. nov. — MycoBank 839589

*Etymology*: the specific epithet *clavatus* refers to the vegetative cell morphology of the type strain.

After 7 days at 17°C in YM broth, cells are ovoid to elongate, cylindrical, club-shaped, 1.3–1.7 × 5.8–16.7 μm and single, a sediment is produced, budding is polar, and hyphae are narrow (1.2–2.5 μm; Figure 7J). After 1 month at 17°C, a ring and a sediment are produced. The streak culture is pale brown, butyrous, and the surface is glistening with slight wrinkle and has an entire margin after 1 month at 17°C on YM agar. Pseudohyphae are formed in Dalmau plate culture on cornmeal agar. Sexual structures are not produced on PDA, YM, CM, and V8 agars. Ballistoconidia are not observed.

Glucose is not fermented. Glucose, galactose (variable), L-sorbose (or latent and weak), sucrose, maltose, cellobiose (variable), trehalose, melibiose, raffinose, melezitose, D-xylose, L-arabinose, D-ribose (variable), ethanol (or latent and weak), erythritol (variable), ribitol (variable), D-mannitol, and D-glucitol are assimilated. Lactose, inulin, soluble starch, D-arabinose, L-rhamnose, D-glucosamine, methanol, glycerol, galactitol, α-Methyl-D-glucoside, salicin (or latent and weak), succinic acid (or weak), DL-lactic acid, citric acid, inositol, and hexadecane are not assimilated. Ammonium sulfate, potassium nitrate, sodium nitrite, L-lysine (variable), ethylamine, and cadaverine are assimilated. Optimal growth is at 17–25°C. Growth does occur in the vitamin-free medium. No starch-like substrate is produced. Growth does not occur on 50% (w/w) glucose-yeast extract agar Urease and Diazonium blue B reactions are positive.

Physiologically, *Y. clavatus* differs from its closely related species, *Y. orchis*, in its inability to assimilate inulin, D-arabinose, and glycerol (Table 2).

*Typus*: China, Fuzhou county, Fujian province, obtained from a leaf of the unidentified plant, August 2011, Qi-Ming Wang, holotype CGMCC 2.4433^T^ preserved in a metabolically inactive state in the CGMCC, Beijing, China. Ex-type CBS 144908 is deposited at the CBS collection of the Westerdijk Fungal Biodiversity Institute, Utrecht, Netherlands; Heilongxiang, Motuo county, Tibet, obtained from a leaf of *Impatiens* sp., September 2014, Qi-Ming Wang, paratype CBS 144917.

##### *Yunzhangomyces qinlingensis* Q.M. Wang, E. Tanaka, M. Groenew. and D. Begerow sp. nov. — MycoBank 839593

*Etymology*: the specific epithet *qinlingensis* refers to the geographic origin of the type strain, Qinling mountain, China.

After 7 days at 17°C in YM broth, cells are cylindrical to elongate, club-shaped, 1.2–1.8 × 6.0–20.8 μm and single, a sediment is produced, budding is polar, and hyphae are narrow, 1.2–2.5 μm (Figure 7K). After 1 month at 17°C, a ring and a sediment are produced. The streak culture is pale yellow, butyrous, the surface is smooth and glistening and has an entire margin after 1 month at 17°C on YM agar. Pseudohyphae and hyphae are absent in Dalmau plate culture on cornmeal agar. Sexual structures are not produced on PDA, YM, CM, and V8 agars. Ballistoconidia are not observed.

Glucose is not fermented. Glucose, galactose, L-sorbose, sucrose, maltose, trehalose, raffinose, melezitose, D-xylose, L-arabinose, D-arabinose (latent and weak), D-ribose, ethanol, erythritol, ribitol (latent and weak), D-mannitol, D-glucitol, α-Methyl-D-glucoside (latent and weak), salicin (latent), succinic acid and citric acid are assimilated. Cellobiose, lactose, melibiose, inulin, soluble starch, L-rhamnose, D-glucosamine, *N*-Acetyl-D-glucosmine, methanol, glycerol, galactitol, DL-lactic acid, inositol, and hexadecane are not assimilated. Ammonium sulfate, potassium nitrate, L-lysine (latent and weak), ethylamine (latent and weak), and cadaverine (latent and weak) are assimilated. Sodium nitrite is not assimilated. Optimal growth is at 17–25°C. Growth does occur in the vitamin-free medium. No starch-like substrate is produced. Growth does not occur on 50% (w/w) glucose-yeast extract agar Urease and Diazonium blue B reactions are positive.

Physiologically, *Y. qinlingensis* differs from its closely related species *Y. cylindricus* in its inability to assimilate cellobiose and inulin and its ability to use melezitose, succinic acid, and citric acid (Table 2).

*Typus*: China, Qinling, Shaanxi province, obtained from a leaf of the unidentified plant, March 2012, Qi-Ming Wang, holotype CGMCC 2.4533^T^ preserved in a metabolically inactive state in the China General Microbiological Culture Collection Center (CGMCC), Beijing, China. Ex-type CBS 144910 is deposited at the CBS collection of the Westerdijk Fungal Biodiversity Institute, Utrecht, Netherlands.

##### *Yunzhangomyces cylindricus* Q.M. Wang, E. Tanaka, M. Groenew. and D. Begerow sp. nov. — MycoBank 839595

*Etymology*: the specific epithet *cylindricus* refers to the vegetative cell morphology of the type strain.

After 7 days at 17°C in YM broth, cells are cylindrical, club-shaped, 1.2–1.8 × 6.0–20.8 μm and single, a sediment is produced, budding is polar, and hyphae are narrow (1.2–2.5 μm; Figure 7L). After 1 month at 17°C, a ring and a sediment are produced. The streak culture is pale yellow, butyrous, and the surface is smooth and glistening and has an entire margin after 1 month at 17°C on YM agar. Pseudohyphae and hyphae are absent in Dalmau plate culture on cornmeal agar. Sexual structures are not produced on PDA, YM, CM, and V8 agars. Ballistoconidia are not observed.

Glucose is not fermented. Glucose, galactose, L-sorbose, sucrose, maltose, cellobiose, trehalose, raffinose, inulin, D-xylose, L-arabinose, D-arabinose, D-ribose, ethanol, erythritol, ribitol, D-mannitol, and D-glucitol are assimilated. Lactose, melibiose, melezitose, soluble starch, L-rhamnose, D-glucosamine, *N*-Acetyl-D-glucosmine, methanol, glycerol, galactitol, α-Methyl-D-glucoside, salicin, DL-lactic acid, succinic acid, citric acid, inositol, and hexadecane are not assimilated. Ammonium sulfate, potassium nitrate, L-lysine, ethylamine, and cadaverine are assimilated. Sodium nitrite is not assimilated. Maximum Optimal growth is at 17–25°C. Growth does occur in the vitamin-free medium. No starch-like substrate is produced. Growth does not occur on 50% (w/w) glucose-yeast extract agar Urease and Diazonium blue B reactions are positive.

Physiologically, *Y. cylindricus* differs from its closely related species *Y. qinlingensis* in its inability to assimilate melezitose, succinic acid, and citric acid and its ability to use cellobiose and inulin (Table 2).

*Typus*: China, Daliangzi river national forest park, Heilongjiang province, obtained from a leaf of the unidentified plant, August 2015, Qi-Ming Wang, holotype CGMCC 2.6304^T^ preserved in a metabolically inactive state in the CGMCC, Beijing, China. Ex-type CBS 15585 is deposited at the CBS collection of the Westerdijk Fungal Biodiversity Institute, Utrecht, Netherlands.

*Note*: All the members of the genus *Yunzhangomyces* were isolated from the leaf of plant, which indicated that they are plant associates.

##### *Guomyces* Q.M. Wang, E. Tanaka, M. Groenew., and D. Begerow *gen.* nov. — MycoBank 839579

*Etymology*: The genus is named in honor of Lin Guo for her pioneering contributions to the taxonomy of smuts.

This genus is proposed for the branch represented by *Meira nicotianae*, which formed a separate branch from the genera in the *Brachybasidiaceae* family (*Exobasidiales*, *Exobasidiomycetes*). The genus is mainly circumscribed by the phylogenetic analysis of the ITS+LSU sequences (Figure 3 and Supplementary Figure 3).

Sexual reproduction is not known. Colonies were butyrous, yellow with eroded margin. Budding cells present or not. Hyphae are formed. Ballistoconidia are not produced.

*Type species*: *Guomyces nicotianae* (H.K. Wang and F.C. Lin) Q.M. Wang, E. Tanaka, M. Groenew., and D. Begerow.

##### *Guomyces nicotianae* (H.K. Wang and F.C. Lin) Q.M. Wang, E. Tanaka, M. Groenew., and D. Begerow, *comb.* nov. — MycoBank 839598

*Basionym*: *Meira nicotianae* H.K. Wang and F.C. Lin, in Cao et al. Phytotaxa 365 (2): 176 (2018).

#### Georgefischeriales (Exobasidiomycetes)

##### *Phragmotaenium parafulvescens* Q.M. Wang, Y.Y. Li, M. Groenew., and M.M. Wang sp. nov. — MycoBank 839586

*Etymology*: the specific epithet, *parafulvescens*, refers to a similar colony morphology to that of *Phragmotaenium fulvescens*.

After 7 days at 17°C in YM broth, cells are cylindrical, 1.0–1.7 × 10.8–25.0 μm and single, a sediment is produced, budding is polar, and hyphae are narrow (1.2–2.5 μm; Figure 7M). After 1 month at 17°C, a ring and a sediment are produced. The streak culture is cream to pale yellow, butyrous, and the surface is glistening with slight wrinkle and has an entire margin after 1 month at 17°C on YM agar. Hyphae are formed in Dalmau plate culture on cornmeal agar. Sexual structures are not produced on PDA, YM, CM, and V8 agars. Ballistoconidia are allantoid or falcate, 1.0–1.6 × 7.5–16.7 μm (Figure 7N).

Glucose is not fermented. Glucose, galactose (latent and weak), L-sorbose (latent and weak), sucrose, maltose, trehalose, Lactose (latent and weak), melibiose (latent and weak), raffinose, melezitose, inulin, soluble starch, D-xylose, L-arabinose, D-arabinose, glycerol, ribitol (latent and weak), D-mannitol, D-glucitol, D-gluenoale, and succinic acid are assimilated. Cellobiose, D-ribose, L-rhamnose, D-glucosamine, *N*-Acetyl-D-glucosmine, methanol, ethanol, erythritol, galactitol, α-Methyl-D-glucoside, salicin, DL-lactic acid, citric acid, inositol, and hexadecane are not assimilated. Ammonium sulfate, potassium nitrate, and L-lysine (latent and weak) are assimilated. Sodium nitrite, ethylamine, and cadaverine are not assimilated. Optimal growth is at 17–25°C. Growth does occur in the vitamin-free medium. No starch-like substrate is produced. Growth does not occur on 50% (w/w) glucose-yeast extract agar Urease and Diazonium blue B reactions are positive.

Physiologically, *P. parafulvescens* differs from its closely related species, *P. fulvescens*, in its inability to assimilate cellobiose, D-ribose, erythritol, citric acid, and sodium nitrite and its positive growth in vitamin-free medium (Table 2).

*Typus*: China, Sanya county, Hainan province, obtained from a leaf of the unidentified plant, May 2007, Qi-Ming Wang, holotype CGMCC 2.3573^T^ preserved in a metabolically inactive state in the CGMCC, Beijing, China. Ex-type CBS 15754 is deposited at the CBS collection of the Westerdijk Fungal Biodiversity Institute, Utrecht, Netherlands.

##### *Gjaerumia pseudominor* Q.M. Wang, Y.Y. Li, M. Groenew., and M.M. Wang sp. nov. — MycoBank 839588

*Etymology*: the specific epithet *pseudominor* refers to the similar colony morphology to that of *Gjaerumia minor*.

After 7 days at 17°C in YM broth, cells are cylindrical or allantoid, 1.2–1.8 × 6.7–22.5 μm and single, a sediment is produced, budding is polar, and hyphae are narrow (1.2–2.5 μm; Figure 7O). After 1 month at 17°C, a sediment is produced. The streak culture is cream to light-yellowish, butyrous, and the surface is glistening with smooth and has an entire margin after 1 month at 17°C on YM agar. Hyphae are formed, narrow and cylindrical in Dalmau plate culture on cornmeal agar. Sexual structures are not produced on PDA, YM, CM, and V8 agars. Ballistoconidia are allantoid or falcate, 1.0–1.6 × 6.7–12.5 μm (Figure 7P).

Glucose is not fermented. Glucose, sucrose, maltose, cellobiose, trehalose, melezitose, solube starch, D-xylose, L-arabinose, glycerol, D-mannitol, D-glucitol, DL-lactic acid, succinic acid, and citric acid are assimilated. Galactose, L-sorbose, lactose, melibiose, raffinose, inulin, D-arabinose, D-ribose, L-rhamnose, D-glucosamine, *N*-Acetyl-D-glucosmine, methanol, ethanol, erythritol, ribitol, galactitol, α-Methyl-D-glucoside, salicin, inositol, and hexadecane are not assimilated. Ammonium sulfate, potassium nitrate, L-lysine, ethylamine, and cadaverine are assimilated. Sodium nitrite is not assimilated. Optimal growth is at 17–25°C. Growth does occur in a vitamin-free medium (weak). No starch-like substrate is produced. Growth does not occur on 50% (w/w) glucose-yeast extract agar Urease and Diazonium blue B reactions are positive.

Physiologically, *G. pseudominor* differs from its closely related species *G. minor* and *G. cyclobalanopsidis* in its inability to assimilate galactose, lactose, melibiose, raffinose, inulin, D-arabinose, and D-ribose (Table 2).

*Typus*: China, Heilongjiang province, obtained from a leaf of the unidentified plant, August 2015, Qi-Ming Wang, holotype CGMCC 2.5616^T^ preserved in a metabolically inactive state in the CGMCC, Beijing, China. Ex-type CBS 144912 is deposited at the CBS collection of the Westerdijk Fungal Biodiversity Institute, Utrecht, Netherlands.

##### *Gjaerumia cyclobalanopsidis* Q.M. Wang, Y.Y. Li, M. Groenew. and M.M. Wang sp. nov. — MycoBank 839591

*Etymology*: the specific epithet *cyclobalanopsidis* refers to *Cyclobalanopsis*, a plant host from which the type strain was isolated.

**FIGURE 8:**
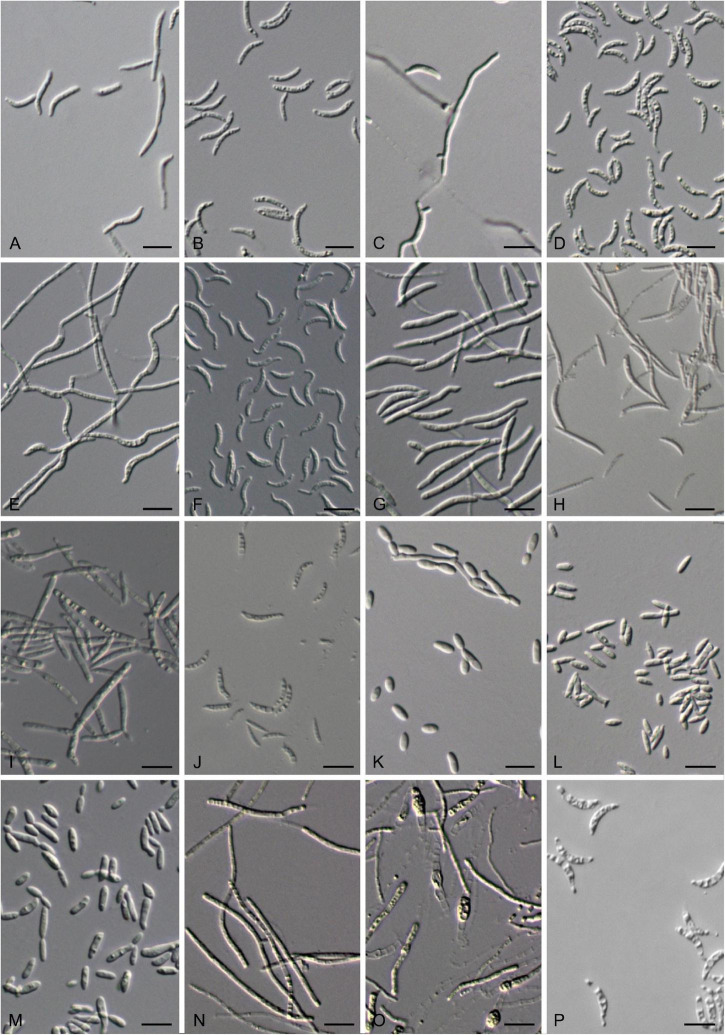
Vegetative cells grown in YM broth for 7 days at 17°C and ballistoconidia produced on cornmeal agar after 7 days at 17°C. **(A,B)** *G. cyclobalanopsidis* CGMCC 2.6419^T^; **(C–F)** *J. aceris* CGMCC 2.5602^T^; **(G,H)** *T. pinicola* CGMCC 2.5613^T^; **(I,J)** *T. lunata* CGMCC 2.6308^T^; **(K)** *J. lantanae* CGMCC 2.3529^T^; **(L)** *S. europaea* CGMCC 2.3119^T^; **(M)** *B. planticola* CGMCC 2.4532^T^; **(N–P)** *F. bambusicola* CGMCC 2.2620^T^. Bars = 10 μm. **(B,D,F,H,P)** are ballistoconidia.

After 7 days at 17°C in YM broth, cells are cylindrical or allantoid, 1.0–1.6 × 7.3–23.6 μm and single, a sediment is produced, budding is polar, and hyphae are narrow (1.2–2.5 μm; Figure 8A). After 1 month at 17°C, a film and sediment are produced. The streak culture is cream to yellowish, butyrous, the surface is wrinkled with gloomy, and has an entire margin after 1 month at 17°C on YM agar. Hyphae are formed, narrow and cylindrical in Dalmau plate culture on cornmeal agar. Sexual structures are not produced on PDA, YM, CM, and V8 agars. Ballistoconidia are allantoid or falcate (1.0–1.7 × 8.3–14.2 μm; Figure 8B).

Glucose is not fermented. Glucose, galactose, L-sorbose (weak), sucrose, maltose, cellobiose, trehalose, lactose, melibiose, raffinose, melezitose, inulin, D-xylose, L-arabinose, D-arabinose, glycerol, ribitol (weak), D-mannitol (weak), and D-glucitol (weak) are assimilated. Solube starch, D-ribose, L-rhamnose, D-glucosamine, *N*-Acetyl-D-glucosmine, methanol, ethanol, erythritol, galactitol, α-Methyl-D-glucoside, salicin, DL-lactic acid, succinic acid, citric acid, inositol, and hexadecane are not assimilated. Ammonium sulfate, potassium nitrate (weak), sodium nitrite, L-lysine (weak), and cadaverine (latent and weak) are assimilated. Ethylamine is not assimilated. Optimal growth is at 17–25°C. Growth does not occur in vitamin-free medium. No starch-like substrate is produced. Growth does not occur on 50% (w/w) glucose-yeast extract agar Urease and Diazonium blue B reactions are positive.

Physiologically, *G. cyclobalanopsidis* differs from its closely related species *G. pseudominor* and *G. minor* in its inability to assimilate soluble starch, DL-lactic acid, and succinic acid (Table 2).

*Typus*: China, Gutianshan, Zhejiang province, obtained from a leaf of *Cyclobalanopsis* sp., June 2011, Qi-Ming Wang, holotype CGMCC 2.6419^T^ preserved in a metabolically inactive state in the CGMCC, Beijing, China. Ex-type CBS 144918 is deposited at the CBS collection of the Westerdijk Fungal Biodiversity Institute, Utrecht, Netherlands.

##### *Jamesdicksonia aceris* Q.M. Wang, Y.Y. Li, M. Groenew. and M.M. Wang sp. nov. — MycoBank MB839779

*Etymology*: the specific epithet *aceris* refers to *Acer*, a plant host from which the type strain was isolated.

After 7 days at 17°C in YM broth, cells are allantoid to elongate, 0.8–1.8 × 7.0–11.8 μm and single, a sediment is produced, budding is polar, hyphae are narrow (1.2–2.5 μm; Figures 8C,E). After 1 month at 17°C, a film and sediment are produced. The streak culture is cream to cream to pink, butyrous, the surface is smooth or slightly wrinkled with glistening, and has an entire margin after 1 month at 17°C on YM agar. Hyphae are formed in Dalmau plate culture on cornmeal agar. Sexual structures are not produced on PDA, YM, CM, and V8 agars. Ballistoconidia are allantoid, falcate, 0.8–1.7 × 7.5–15.8 μm (Figures 8D,F).

Glucose is not fermented. Glucose, galactose (variable), sucrose, maltose, cellobiose, trehalose, lactose (variable), raffinose (or weak), melezitose (or weak), inulin (variable), soluble starch (variable), D-xylose (variable), L-arabinose, D-arabinose, D-ribose, L-rhamnose, glycerol, erythritol, ribitol, D-mannitol, D-glucitol, and succinic acid (variable) are assimilated. L-sorbose, melibiose, D-glucosamine, methanol, ethanol, galactitol, α-Methyl-D-glucoside, salicin, DL-lactic acid, citric acid, inositol, and hexadecane are not assimilated. Ammonium sulfate, potassium nitrate, L-lysine, ethylamine, and cadaverine are assimilated. Sodium nitrite is not assimilated. Optimal growth is at 17–25°C. Growth does occur in the vitamin-free medium. No starch-like substrate is produced. Growth does not occur on 50% (w/w) glucose-yeast extract agar Urease and Diazonium blue B reactions are positive.

Physiologically, *J. aceris* differs from its closely related species *J. mali* in its inability to assimilate melibiose, D-glucosamine, and sodium nitrite and its ability to use D-arabinose, ribitol, and cadaverine (Table 2).

*Typus*: China, Bomi, Tibet, obtained from a leaf of *Acer pectinatum*, September 2014, Qi-Ming Wang, holotype CGMCC 2.5602^T^ preserved in a metabolically inactive state in the CGMCC, Beijing, China. Ex-type CBS 144916 is deposited at the CBS collection of the Westerdijk Fungal Biodiversity Institute, Utrecht, Netherlands. Bomi, Tibet, obtained from a leaf of the unidentified plant, September 2014, Qi-Ming Wang, paratype XZ156C4. Heilongjiang province, obtained from a leaf of an unidentified plant, Aug. 2015, Qi-Ming Wang, paratype CGMCC 2.5679. Jilin province, Changbai mountain, obtained from a leaf of the unidentified plant, Oct. 1998, Feng-Yan Bai, paratype CGMCC 2.2370.

#### Entylomatales (Exobasidiomycetes)

##### *Tilletiopsis pinicola* Q.M. Wang, Y.Y. Li, M. Groenew. and M.M. Wang sp. nov. — MycoBank 839596

*Etymology*: the specific epithet *pinicola* refers to *Pinus* plant host from which the type strain was isolated.

After 7 days at 17°C in YM broth, cells are cylindrical, 1.3–2.7 × 6.7–28.3 μm and single, a sediment is produced, budding is polar, and hyphae are narrow (1.2–2.5 μm; Figure 8G). After 1 month at 17°C, a film and sediment are produced. The streak culture is pale yellow, butyrous, the surface is glistening with slightly granular, and has an entire margin after 1 month at 17°C on YM agar. Pseudohyphae are formed in Dalmau plate culture on cornmeal agar. Sexual structures are not produced on PDA, YM, CM, and V8 agars. Ballistoconidia are lunate to sickle-shaped or cylindrical (0.8–1.3 × 8.3–19.1 μm; Figure 8H).

Glucose is not fermented. Glucose, galactose, sucrose, maltose, trehalose, melibiose (weak), raffinose, melezitose, D-xylose, L-arabinose, D-arabinose, D-ribose, ethanol, glycerol, erythritol, D-glucitol (weak), succinic acid (weak), and citric acid (weak) are assimilated. L-sorbose, cellobiose, lactose, inulin, soluble starch, L-rhamnose, D-glucosamine, *N*-Acetyl-D-glucosmine, methanol, ribitol, galactitol, D-mannitol, α-Methyl-D-glucoside, salicin, DL-lactic acid, inositol, and hexadecane are not assimilated. Ammonium sulfate, potassium nitrate, L-lysine, ethylamine, and cadaverine are assimilated. Sodium nitrite is not assimilated. Optimal growth is at 17–25°C. Growth does occur in the vitamin-free medium. No starch-like substrate is produced. Growth does not occur on 50% (w/w) glucose-yeast extract agar Urease and Diazonium blue B reactions are positive.

Physiologically, *T. pinicola* differs from its closely related species, *T. lilacina*, in its inability to assimilate cellobiose, soluble starch, ribitol, D-mannitol, α-Methyl-D-glucoside, DL-lactic acid, and sodium nitrite and its ability to assimilate ethanol and grow in the vitamin-free medium (Table 2).

*Typus*: China, Heilongjiang province, obtained from a leaf of *Pinus* sp., August 2015, Qi-Ming Wang, holotype CGMCC 2.5613^T^ preserved in a metabolically inactive state in the CGMCC, Beijing, China. Ex-type CBS 15775 is deposited at the CBS collection of the Westerdijk Fungal Biodiversity Institute, Utrecht, Netherlands.

##### *Tilletiopsis lunata* Q.M. Wang, Y.Y. Li, M. Groenew. and M.M. Wang sp. nov. — MycoBank 839594

*Etymology*: the specific epithet, *lunata*, refers to the vegetative cell morphology of the type strain.

After 7 days at 17°C in YM broth, cells are cylindrical, 1.0–2.5 × 10.0–25.2 μm and single, a sediment is produced, budding is polar, and hyphae are narrow (1.2–2.5 μm; Figure 8I). After 1 month at 17°C, an easy-dispersed film and sediment are produced. The streak culture is pale-yellowish, butyrous, flat, and the surface is venous and has an entire margin after 1 month at 17°C on YM agar. Hyphae are formed in Dalmau plate culture on cornmeal agar. Sexual structures are not produced on PDA, YM, CM, and V8 agars. Ballistoconidia are lunate to sickle-shaped (0.8–2.1 × 8.3–21.6 μm; Figure 8J).

Glucose is not fermented. Glucose, galactose, sucrose, maltose, trehalose, raffinose, melezitose, inulin, soluble starch, D-xylose, L-arabinose, D-ribose, ethanol, glycerol, erythritol, ribitol (variable), D-mannitol, D-glucitol, succinic acid, and citric acid are assimilated. L-sorbose, cellobiose, lactose, D-arabinose, L-rhamnose, D-glucosamine, *N*-Acetyl-D-glucosmine, methanol, galactitol, α-Methyl-D-glucoside, salicin, D-glueonale, DL-lactic acid, inositol, and hexadecane are not assimilated. Ammonium sulfate, potassium nitrate, sodium nitrite (variable), L-lysine, and cadaverine are assimilated. Ethylamine is not assimilated. Optimal growth is at 17–25°C. Growth does occur in the vitamin-free medium. No starch-like substrate is produced. Growth does not occur on 50% (w/w) glucose-yeast extract agar Urease and Diazonium blue B reactions are positive.

Physiologically, *T. lunata* differs from its closely related species, *T. washingtonensis*, in its inability to assimilate D-arabinose, D-glueonale, and DL-lactic acid and its ability to use inulin, and its positive growth in the vitamin-free medium (Table 2).

*Typus*: China, Huzhong, Heilongjiang Province, obtained from a leaf of the unidentified plant, August 2015, Qi-Ming Wang, holotype CGMCC 2.6308^T^ preserved in a metabolically inactive state in the CGMCC, Beijing, China. Ex-type DSM 111865 is deposited at the German Collection of Microorganisms and Cell Cultures, Braunschweig, Germany. Huzhong, Heilongjiang Province, obtained from a leaf of the unidentified plant, August 2015, Qi-Ming Wang, paratype CGMCC 2.6307.

*Note*: The two new species of *Tilletiopsis* and the known species, *T. cremea* and *T. lilacina*, are all isolated from plant leaves (Boekhout et al., 2011). *T. washingtonensis* seems to be a common inhabitant of the phyllosphere, but it also occurs in the soil, rotten wood, and tree bark (Supplementary Table 1).

#### Microstromatales (Exobasidiomycetes)

##### *Jaminaea lantanae* Q.M. Wang, Y.Y. Li, M. Groenew. and M.M. Wang sp. nov. — MycoBank 839590

*Etymology*: the specific epithet *lantanae* refers to *Lantana* plant host, from which the type strain was isolated.

After 7 days at 17°C in YM broth, cells are ovoid to elongate, ellipsoidal, 1.7–2.9 × 3.3–10.0 μm and single, a sediment is produced, budding is polar, and hyphae are narrow (1.2–2.5 μm; Figure 8K). After 1 month at 17°C, an easy-dispersed film and sediment are produced. The streak culture is pink, butyrous, flat, the surface is wrinkled and moist with glistening, and has an entire margin after 1 month at 17°C on YM agar. Hyphae are absent in Dalmau plate culture on cornmeal agar. Sexual structures are not produced on PDA, YM, CM, and V8 agars. Ballistoconidia are not observed.

Glucose is not fermented. Glucose, L-sorbose, sucrose (latent), maltose (latent and weak), cellobiose (latent and weak), melezitose (latent and weak), D-xylose (latent), L-arabinose, D-glucosamine (latent), *N*-Acetyl-D-glucosmine, ethanol, glycerol, erythritol, D-mannitol (latent), D-glucitol (latent and weak), α-Methyl-D-glucoside (latent and weak), and salicin (latent and weak) are assimilated. Galactose, trehalose, melibiose, raffinose, lactose, inulin, soluble starch, D-arabinose, D-Ribose, L-rhamnose, methanol, ribitol, galactitol, D-glueonale, DL-lactic acid, succinic acid, citric acid, inositol, and hexadecane are not assimilated. Ammonium sulfate, L-lysine, ethylamine, and cadaverine are assimilated. Potassium nitrate and sodium nitrite are not assimilated. Optimal growth is at 17–25°C. Growth does occur in the vitamin-free medium. No starch-like substrate is produced. Growth does not occur on 50% (w/w) glucose-yeast extract agar Urease and Diazonium blue B reactions are positive.

*Typus*: China, Haikou county, Hainan province, obtained from a leaf of *Lantana camara*, Aug. 2007, Qi-Ming Wang, holotype CGMCC 2.3529^T^ preserved in a metabolically inactive state in the CGMCC, Beijing, China. Ex-type CBS 15493 is deposited at the CBS collection of the Westerdijk Fungal Biodiversity Institute, Utrecht, Netherlands. Haikou county, Hainan province, obtained from a leaf of *Lantana camara*, Aug. 2007, Qi-Ming Wang, paratype CGMCC 2.3622.

*Note*: *J. angkorensis* was isolated from fallen decaying leaves in Cambodia, *J. lanaiensis* from marine driftwood in Hawaiian, *J. pallidilutea* from plant material from mangrove in Iran, *J. rosea* from phylloplane of *Plumeria* in Florida (Sipiczki and Kajdacsi, 2009; Wei et al., 2011; Kijpornyongpan and Aime, 2017; Nasr et al., 2017). *J. lantanae* was isolated from the leaf of *Lantana camara*. The above data indicated that the members of *Jaminaea* are widespread along with plant associates.

##### *Sympodiomycopsis europaea* Q.M. Wang, Y.Y. Li, M. Groenew. and F.Y. Bai sp. nov. — MycoBank 839583

*Etymology*: the specific epithet, *europaea*, refers to the geographic origin of the type strain, Europe.

After 7 days at 17°C in YM broth, cells are or ellipsoidal to elongate, cylindrical, 1.5–1.8 × 4.2–12.5 μm and single, a sediment is produced, budding is polar, hyphae are narrow (1.2–2.5 μm; Figure 8L). After 1 month at 17°C, a ring and a sediment are produced. The streak culture is pink colored, butyrous, flat, and the surface is rachnoid with dull and has an entire margin after 1 month at 17°C on YM agar. Pseudohyphae are formed in Dalmau plate culture on cornmeal agar. Sexual structures are not produced on PDA, YM, CM, and V8 agars. Ballistoconidia are not observed.

Glucose is not fermented. Glucose, galactose, L-sorbose, sucrose, maltose, trehalose (latent and weak), lactose, melibiose, raffinose, melezitose, soluble starch (weak), D-xylose, L-arabinose (or latent and weak), D-ribose, ethanol, glycerol, erythritol, D-mannitol, D-glucitol (or latent and weak), and α-Methyl-D-glucoside (or latent and weak) are assimilated. Cellobiose, inulin, D-arabinose, L-rhamnose, D-glucosamine, methanol, ribitol, galactitol, salicin, DL-lactic acid, succinic acid (or weak), citric acid (or latent and weak), inositol (or weak), and hexadecane are not assimilated. Ammonium sulfate, potassium nitrate (variable), L-lysine (weak), and cadaverine are assimilated. Sodium nitrite and ethylamine are not assimilated. Optimal growth is at 17–25°C. Growth does occur in the vitamin-free medium. No starch-like substrate is produced. Growth does not occur on 50% (w/w) glucose-yeast extract agar Urease and Diazonium blue B reactions are positive.

Physiologically, *S. europaea* differs from its closely related species, *S. kandeliae* and *S. paphiopedili*, in its inability to assimilate cellobiose, D-arabinose, and grow in the 50% glucose medium (Table 2).

*Typus*: Germany, obtained from a leaf of the unidentified plant, March 2006, Feng-Yan Bai, holotype CGMCC 2.3119^T^ preserved in a metabolically inactive state in the CGMCC, Beijing, China. Ex-type CBS 15470 is deposited at the CBS collection of the Westerdijk Fungal Biodiversity Institute, Utrecht, Netherlands; Obtained from a leaf of the unidentified plant, Mar. 2006, Feng-Yan Bai, paratypes CGMCC 2.3181, CGMCC 2.3120, CGMCC 2.3121, CGMCC 2.3122, CGMCC 2.3123 and CGMCC 2.3124.

*Note*: The species of *Sympodiomycopsis* seem to associate with plant substrate. *S. paphiopedili* was isolated from the nectar of *Paphiopedilum primulinum*, *S. kandeliae* from flowers of *Kandelia candel* in the mangrove forest, *S. yantaiensis* from insect frass from the trunk of *Sophora japonica*, and the newly described species *S. europaea* also from the leaf of plant.

##### *Baueromyces* Q.M. Wang, D. Begerow, and M. Groenew. *gen.* nov. — MycoBank 839577

*Etymology*: the genus is named in honor of Robert Bauer for his pioneering work on the taxonomy of smuts.

This genus is proposed for the branch represented by strain CGMCC 2.4532, which formed a separate branch from the genera in the *Microstromatales* (*Exobasidiomycetes*). The genus is mainly circumscribed by the phylogenetic analysis of the six-gene sequences (Figure 1).

Sexual reproduction not known. Colonies are butyrous, pink, and have smooth margins. Budding cells are present. Ballistoconidia are not produced. Hyphae are not formed.

*Type species*: *Baueromyces planticola* Q.M. Wang, D. Begerow and M. Groenew.

##### *Baueromyces planticola* Q.M. Wang, D. Begerow, and M. Groenew. sp. nov. — MycoBank 839597

*Etymology*: the specific epithet, *planticola*, refers to the substrate origin of the type strain, plant.

After 7 days at 17°C in YM broth, cells are cylindrical, 1.7–2.5 × 4.2–10.0 μm and single, a sediment is produced, budding is polar, hyphae are narrow (1.2–2.5 μm; Figure 8M). After 1 month at 17°C, a film or ring and a sediment are produced. The streak culture is cream to pink, butyrous, flat, and the surface is smooth or slightly wrinkled with glistening and has an entire margin after 1 month at 17°C on YM agar. Pseudohyphae and Hyphae are absent in Dalmau plate culture on cornmeal agar. Sexual structures are not produced on PDA, YM, CM, and V8 agars. Ballistoconidia are not observed.

Glucose is not fermented. Glucose, galactose, sucrose, maltose (week), cellobiose, trehalose (latent and weak), lactose (weak), melibiose (variable), raffinose, melezitose (variable), inulin, D-xylose (weak), L-arabinose, D-ribose (variable), L-rhamnose (variable), *N*-Acetyl-D-glucosmine (variable), glycerol, erythritol (latent and weak), D-mannitol, and D-glucitol are assimilated. L-sorbose, soluble starch, D-arabinose, D-glucosamine, methanol, ethanol (or weak), ribitol, galactitol, α-Methyl-D-glucoside (weak), salicin (or latent and weak), DL-lactic acid, succinic acid (or weak), citric acid, inositol (or latent and weak), and hexadecane are not assimilated. Ammonium sulfate, potassium nitrate (or weak), L-lysine (or weak), ethylamine (or weak), and cadaverine (or weak) are assimilated. Sodium nitrite (or latent and weak) is not assimilated. The maximum growth temperature is 30–32°C. Growth does occur in the vitamin-free medium. No starch-like substrate is produced. Growth does not occur on 50% (w/w) glucose-yeast extract agar Urease and Diazonium blue B reactions are positive.

*Typus*: China, Xingshan County, Hubei province, obtained from a leaf of the unidentified plant, March 2012, Qi-Ming Wang, holotype CGMCC 2.4532^T^ preserved in a metabolically inactive state in the CGMCC, Beijing, China. Ex-type CBS 144909 is deposited at the CBS collection of the Westerdijk Fungal Biodiversity Institute, Utrecht, Netherlands. Maotai county, Guizhou province, obtained from a leaf of the unidentified plant, March 2012, Qi-Ming Wang, CGMCC 2.4534. Fanjingshan, Guizhou province, obtained from a leaf of the unidentified plant, March 2012, Qi-Ming Wang, paratypes CGMCC 2.4535 and CGMCC 2.4536.

##### *Franziozymales* Q.M. Wang, D. Begerow, and M. Groenew. *ord.* nov. — MycoBank 839576

Member of *Exobasidiomycetes*. The diagnosis of the order *Franziozymales* is based on the description of the genus *Franziozyma*. The nomenclature of the order is based on the genus *Franziozyma*.

*Type family*: *Franziozymaceae* Q.M. Wang, D. Begerow, M. Groenew.

##### *Franziozymaceae* Q.M. Wang, D. Begerow and M. Groenew. *fam.* nov. — MycoBank 839575

Member of *Franziozymales* (*Exobasidiomycetes*). The diagnosis of the family *Franziozymaceae* is based on the description of the genus, *Franziozyma*. The nomenclature of the family is based on the genus.

*Type genus*: *Franziozyma* Q.M. Wang, D. Begerow and M. Groenew.

*Genus accepted*: *Franziozyma* Q.M. Wang, D. Begerow and M. Groenew.

##### *Franziozyma* Q.M. Wang, D. Begerow, and M. Groenew. *gen.* nov. — MycoBank 839574

*Etymology*: the genus is named in honor of Franz Oberwinkler for his pioneering work on the taxonomy of smuts.

This genus is proposed for the branch represented by strain XZ4C4^T^, which formed a separate branch from *Golubeviales* and other orders in *Exobasidiomycetes*. The genus is mainly circumscribed by the phylogenetic analysis of the six loci dataset (Figure 1).

Sexual reproduction is not known. Colonies are butyrous, white, margin, or eroded. Budding cells present or not. Hyphae are formed. Ballistoconidia are produced.

*Type species*: *Franziozyma bambusicola* Q.M. Wang, D. Begerow and M. Groenew.

##### *Franziozyma bambusicola* Q.M. Wang, D. Begerow and M. Groenew. sp. nov. — MycoBank 839573

*Etymology*: the specific epithet *bambusicola* refers to the origin of the substrate of the type strain of this species.

After 10 days at 17°C on 5% malt extract agar, colonies are cream, soft or tough, usually glabrous, or sometimes pubescent, shiny or dull, ridged, and with an eroded margin. Hyphae are narrow and cylindrical, usually 1.0–1.3 × 30–60 μm, sometimes regularly branched (Figure 8N). Chlamydospores occur intercalarily or terminally and are single, subglobose or ellipsoidal, 4.0–6.0 × 6–10 μm (Figure 8O). Sexual structures are not produced on YM, YPD, V8, and CM agars. Ballistoconidia are ellipsoidal orallantoid, 2.3–2.7 × 13.6–18.2 μm (Figure 8P).

Glucose is not fermented. Glucose, sucrose, maltose, cellobiose, trehalose, raffinose, inulin, soluble starch, glycerol, erythritol, D-mannitol, and D-glucitol are assimilated. Galactose, L-sorbose, lactose, melibiose, melezitose, D-xylose, L-arabinose, D-arabinose, D-ribose, L-rhamnose, D-glucosamine, methanol, ethanol, ribitol, galactitol, α-Methyl-D-glucoside, salicin, DL-lactate, and succinate, citrate, myo-inositol, and hexadecane are not assimilated. The maximum growth temperature is 22°C. Growth in the vitamin-free medium is weak. No starch-like substrate is produced. Growth does not occur on 50% (w/w) glucose-yeast extract agar Urease and Diazonium blue B reactions are positive.

*Typus*: China, Bomi County, Tibet, obtained from a leaf of bamboo, Sep. 2004, Qi-Ming Wang, holotype CGMCC 2.2620^T^ preserved in a metabolically inactive state in the CGMCC, Beijing, China. Ex-type CBS 15774 is deposited at the CBS collection of the Westerdijk Fungal Biodiversity Institute, Utrecht, Netherlands. Bomi county, Tibet, obtained from a leaf of bamboo, September 2004, Qi-Ming Wang, paratype XZ4A1.

## Data Availability Statement

The datasets presented in this study can be found in online repositories. The names of the repository/repositories and accession number(s) can be found in the article/Supplementary Material.

## Author Contributions

Q-MW conceived and designed the project. Q-MW, Y-YL, Y-TG, and FW performed sampling and yeast isolation. Y-YL, A-HL, M-MW, and Q-MW performed phenotypic characterization and analyzed the molecular data. B-QZ registered the taxa in MycoBank submitted the sequence data in TreeBASE. Q-MW, M-MW, MG, and DB wrote the manuscript. ET and F-YB revised the manuscript. ET supported the sequences generated in his laboratory. All authors contributed to the article and approved the submitted version.

## Conflict of Interest

The authors declare that the research was conducted in the absence of any commercial or financial relationships that could be construed as a potential conflict of interest.

## Publisher’s Note

All claims expressed in this article are solely those of the authors and do not necessarily represent those of their affiliated organizations, or those of the publisher, the editors and the reviewers. Any product that may be evaluated in this article, or claim that may be made by its manufacturer, is not guaranteed or endorsed by the publisher.

## Supplementary Material

Supplementary Figure 1 Phylogeny of new yeast or yeast-like species in the *Exobasidiomycetes* inferred from the sequences of the LSU rDNA D1/D2 domains and ITS region (including 5.8S rDNA) by maximum likelihood analysis and over 50% from 1,000 bootstrap replicates is shown. Bar = 0.1 substitutions per nucleotide position. The compressed genera are monophyletic, the species in those clades were listed in Table 1 and Supplementary Table 2.

Supplementary Figure 2 Phylogeny of new yeast or yeast-like species in the *Exobasidiomycetes* inferred from the sequences of the LSU rDNA D1/D2 domains by maximum likelihood analysis and over 50% from 1,000 bootstrap replicates is shown. Bar = 0.05 and 0.02 substitutions per nucleotide position. The compressed genera are monophyletic, the species in those clades were listed in Table 1 and Supplementary Table 2.

Supplementary Figure 3 Phylogeny of new yeast or yeast-like species in the *Exobasidiomycetes* inferred from the sequences of the LSU rDNA D1/D2 domains by maximum-likelihood analysis and over 50% from 1000 bootstrap replicates is shown. Bar = 0.05 and 0.02 substitutions per nucleotide position. The compressed genera are monophyletic, the species in those clades were listed in Table 1 and Supplementary Table 2.
