# Supplementary figures and images for: Proposal of Two New Combinations, Twenty New Species, Four New Genera, One New Family, and One New Order for the Anamorphic Basidiomycetous Yeast Species in Ustilaginomycotina
Source: Front Microbiol. 2022 Feb 11;12:777338. doi: 10.3389/fmicb.2021.777338 (PMC8880017; doi:10.3389/fmicb.2021.777338)

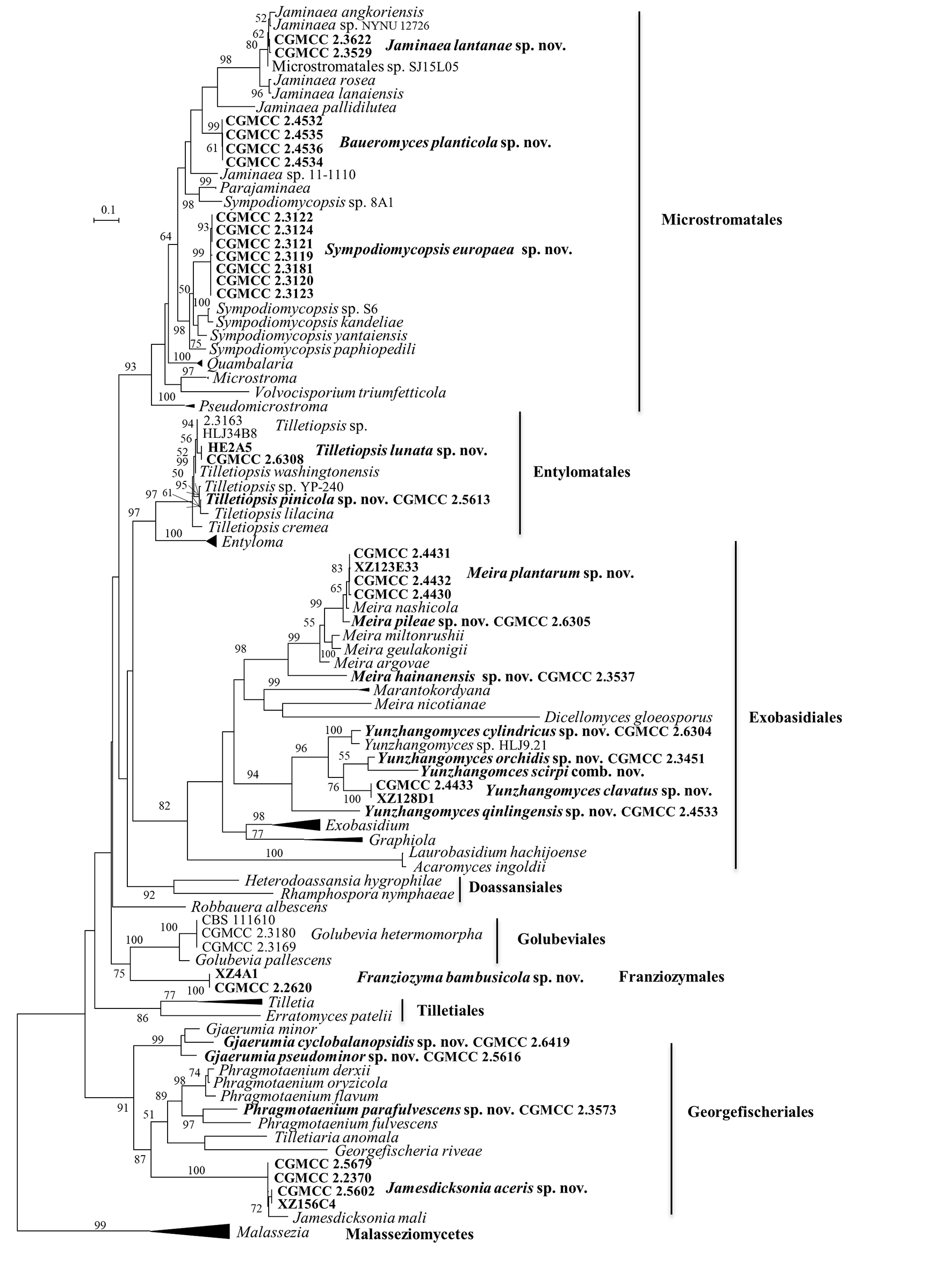

Supplement: Supplementary Figure 1 — Phylogeny of new yeast or yeast-like species in the Exobasidiomycetes inferred from the sequences of the LSU rDNA D1/D2 domains and ITS region (including 5.8S rDNA) by maximum likelihood analysis and over 50% from 1,000 bootstrap replicates is shown. Bar = 0.1 substitutions per nucleotide position. The compressed genera are monophyletic, the species in those clades were listed in Table 1 and Supplementary Table 2. [file Image_1.TIF]

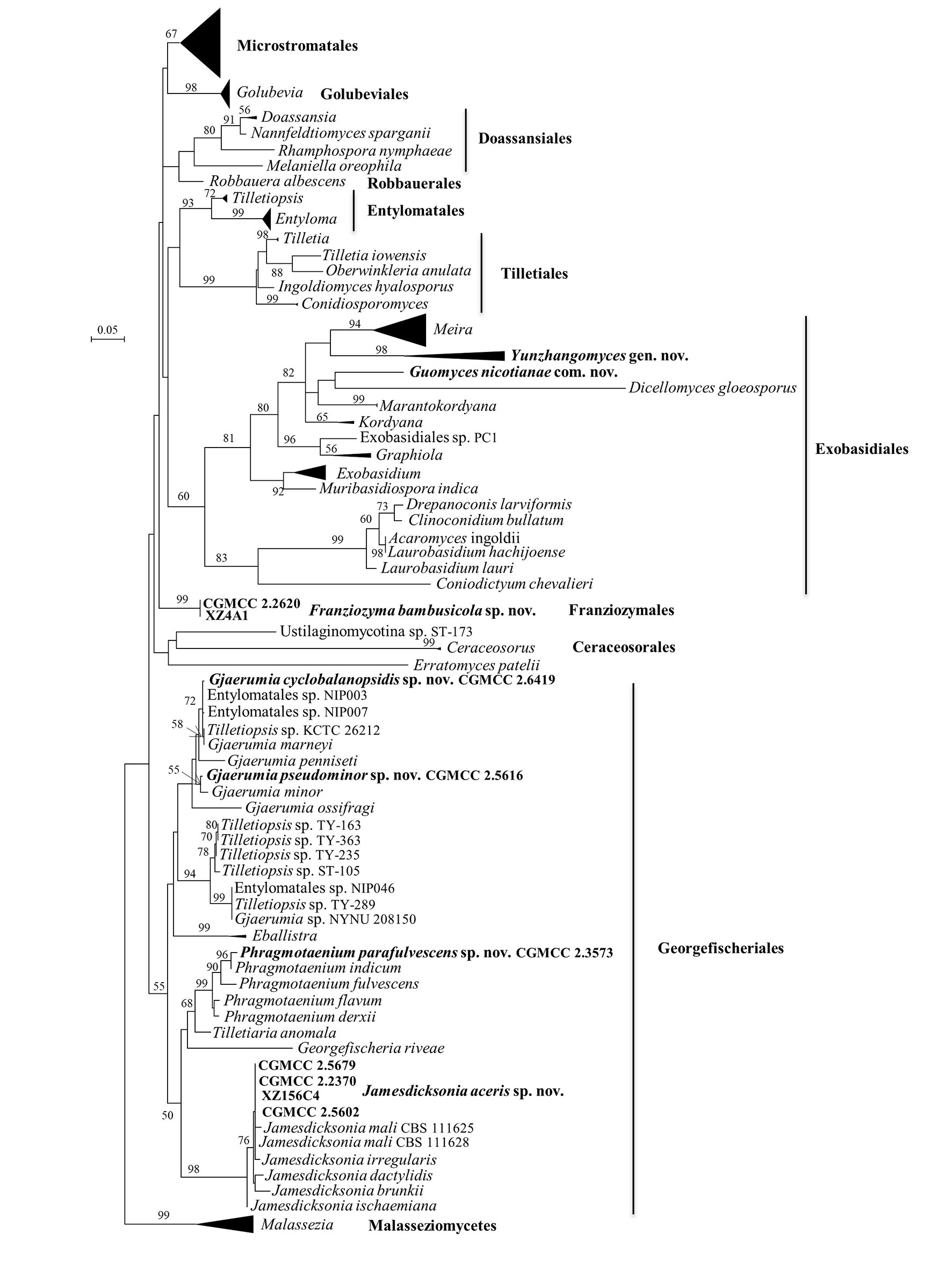

Supplement: Supplementary Figure 2 — Phylogeny of new yeast or yeast-like species in the Exobasidiomycetes inferred from the sequences of the LSU rDNA D1/D2 domains by maximum likelihood analysis and over 50% from 1,000 bootstrap replicates is shown. Bar = 0.05 and 0.02 substitutions per nucleotide position. The compressed genera are monophyletic, the species in those clades were listed in Table 1 and Supplementary Table 2. [file Image_2.TIF]

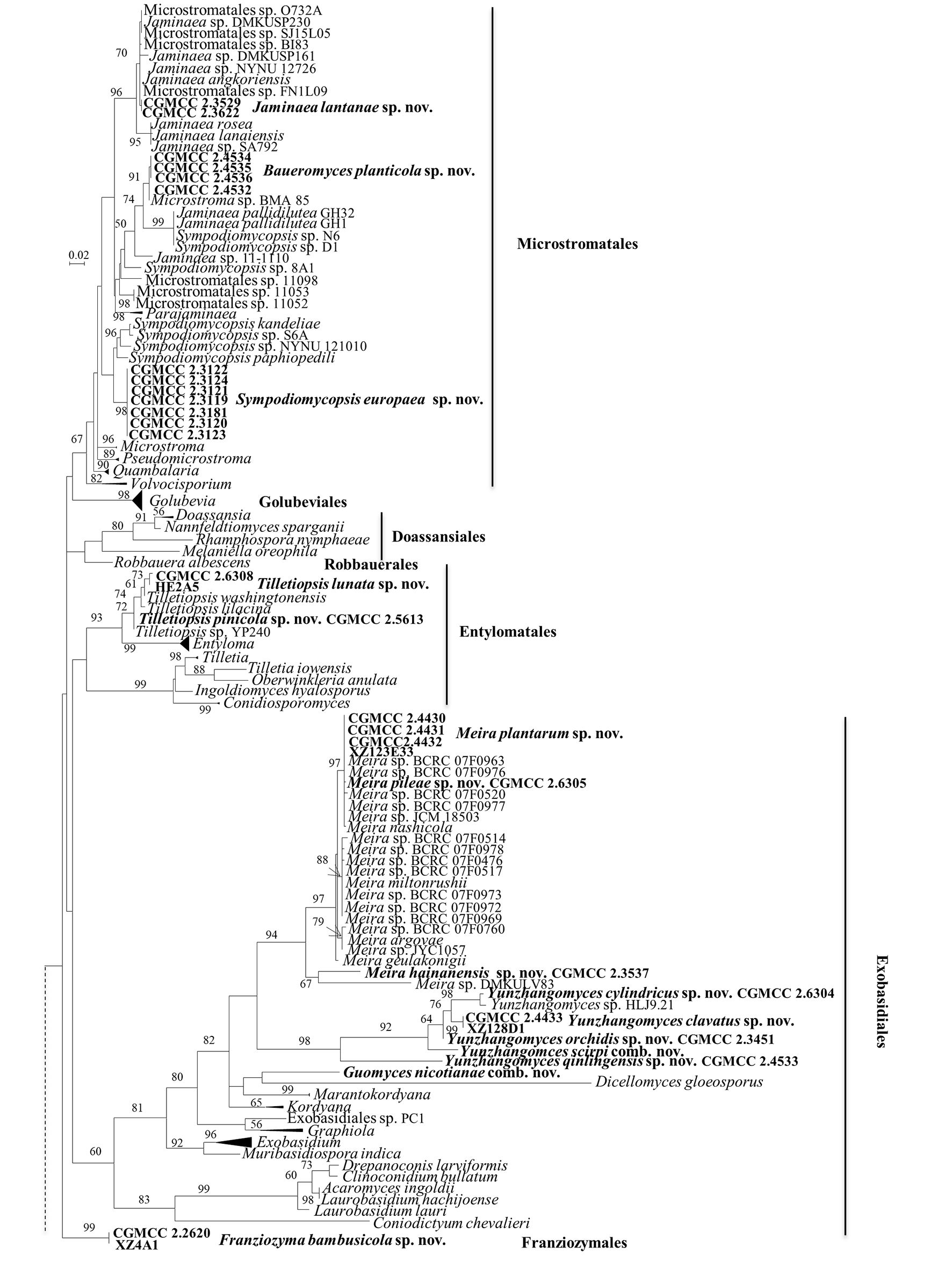

Supplement: Supplementary Figure 3 — Phylogeny of new yeast or yeast-like species in the Exobasidiomycetes inferred from the sequences of the LSU rDNA D1/D2 domains by maximum-likelihood analysis and over 50% from 1000 bootstrap replicates is shown. Bar = 0.05 and 0.02 substitutions per nucleotide position. The compressed genera are monophyletic, the species in those clades were listed in Table 1 and Supplementary Table 2. [file Image_3.TIF]
